# MiRNA-671-5p Promotes prostate cancer development and metastasis by targeting NFIA/CRYAB axis

**DOI:** 10.1038/s41419-020-03138-w

**Published:** 2020-11-03

**Authors:** Zhiguo Zhu, Lianmin Luo, Qian Xiang, Jiamin Wang, Yangzhou Liu, Yihan Deng, Zhigang Zhao

**Affiliations:** Department of Urology & Andrology, Minimally Invasive Surgery Center, Guangdong Provincial Key Laboratory of Urology, The First Affiliated Hospital of Guangzhou Medical University. Guangzhou, Guangdong, China

**Keywords:** Targeted therapies, Prostate cancer, Prognostic markers, Prostate cancer

## Abstract

Prostate cancer (PCa) is the second cause of death due to malignancy among men, and metastasis is the leading cause of mortality in patients with PCa. MicroRNAs (miRNAs) play important regulatory roles in tumor development and metastasis. Here, we identified 13 miRNAs related to PCa metastasis by bioinformatics analysis. Moreover, we found that miR-671-5p was increased in metastatic PCa tissues, and its high expression indicated poor prognosis of PCa. MiR-671-5p could facilitate PCa cells proliferation, migration, and invasion in vitro and vivo. We confirmed that miR-671-5p directly bound to the 3’ untranslated regions of NFIA mRNA, and NFIA directly bound to the CRYAB promoter. High expression of NFIA and CRYAB negatively correlated with the advanced clinicopathological characteristics and metastasis status of PCa patients. Our study demonstrated that miR-671-5p promoted PCa development and metastasis by suppressing NFIA/ CRYAB axis.

## Introduction

Prostate cancer (PCa) is the most frequently diagnosed cancer and the second cause of death due to malignancy among men^1^. In 2020, PCa alone accounts for 21% new diagnosed cancer in men in United States^2^. Thanks to the tremendous progress of systemic and individualized treatments of PCa in the last decades, patients with localized disease have a better prognosis outcome. But metastasis, which is the leading cause of mortality in PCa patients, is still a huge challenge^3^. Therefore, identifying the causes and molecular mechanism of metastasis is important for early detection, diagnosis and personalized therapy.

MicroRNAs (miRNAs) are endogenous non-coding small RNA, which play an important post-transcriptional regulatory role by targeting downstream mRNAs^4^. Accumulating studies have suggested that miRNAs play important roles in many biological processes, including the initiation and progression of cancer. Although many miRNAs, such as miR-34a^5^, miR-532-3p^6^, miR-210-3p^7^, and miR-30d^8^, have been confirmed to promote or suppress metastasis in PCa, there are still many other miRNAs that play important regulatory roles in metastasis that have not yet been discovered. Therefore, we hope to identify miRNAs related to PCa metastasis through bioinformatics analysis, and clarify the molecular mechanism of the identified miRNA in PCa progress.

Here we identified 13 miRNAs related to PCa metastasis, which may play a key regulatory role in the metastasis process. MiR-671-5p (MiR-671) expression was elevated in PCa tissues compared with adjacent normal tissues (ANT). Importantly, the expression levels of miR-671 increased steadily from ANT, primary localized PCa tissues (PPCa), to metastatic PCa tissues (MPCa), and high expression of miR-671 indicated poor prognosis. Overexpression of miR-671 promoted, while knockdown of miR-671 inhibited the proliferation, migration, and invasion of PCa cells in vitro. Furthermore, silencing miR-671 significantly suppressed the proliferation and metastasis of PC-3 cells in vivo. NFIA, a transcription factor and novel tumor suppressor gene, and CRYAB, a member of small heat shock protein family, were direct and indirect downstream targets of miR-671. High expression of NFIA and CRYAB negatively correlated with the advanced clinicopathological characteristics and metastasis status of PCa patients. MiR-671/NFIA/CRYAB axis might be novel therapeutic targets or prognostic markers for PCa.

## Results

### Identifying miRNAs related to PCa metastasis by bioinformatics analysis

**Fig. 1:**
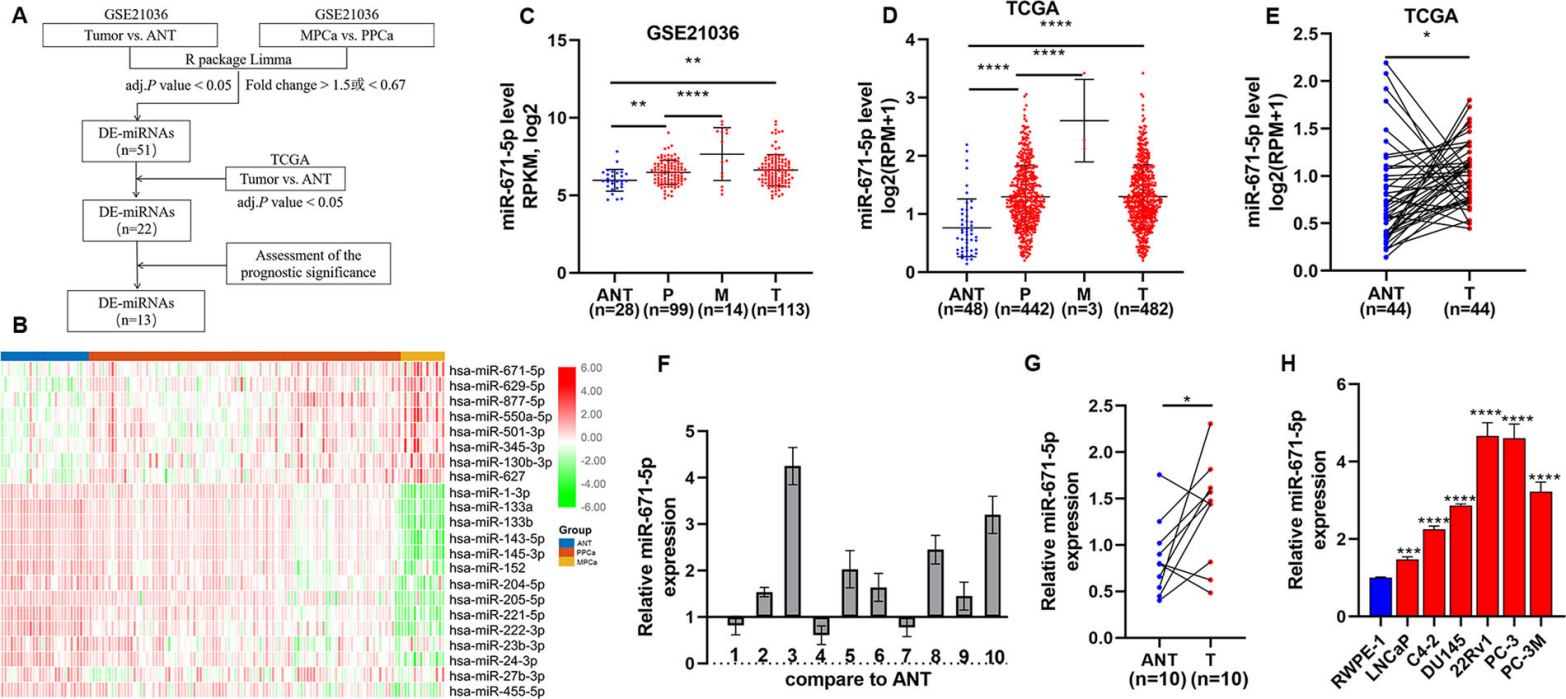
MiR-671 was upregulated in metastatic PCa tissues. **A** The flow chart of bioinformatics analysis for screening miRNAs associated with PCa metastasis. **B** The expression profile of 22 DE-miRNAs in GSE21036. **C**, **D** The expression levels of miR-671 increased steadily from adjacent normal tissues, primary localized PCa tissues, to metastatic PCa tissues in GSE21036 and TCGA. **E** MiR-671 expression levels was upregulated in 44 paired PCa tissues compared with that in the matching adjacent normal tissues in TCGA. **F**, **G** Real-time PCR analysis of miR-671 expression in 10 paired PCa tissues. **H** Real-time PCR analysis of miR-671 expression in normal prostate epithelial cell (RWPE-1) and 6 PCa cells. The data were presented as means ± SD from three biological replicates. *P < 0.05; **P < 0.01; ***P < 0.001; ****P < 0.0001; Student’s *t*-test (**C**, **D**, **H**); paired *t*-test (**E**, **G**). ANT, adjacent normal tissues; P or PPCa, primary localized PCa tissues; M or MPCa, metastatic PCa tissues; T, tumor tissues; DE-miRNAs, differentially expressed miRNAs.

The flow chart of bioinformatics analysis was shown in Fig. 1A. First, we identified differentially expressed miRNAs (DE-miRNAs) related to PCa metastasis in GSE21036. A total of 51 DE-miRNAs (27 upregulated and 24 downregulated) were detected (Table S1). Then, we used TCGA-PRAD data to further narrow the scope of analysis, and 22 DE-miRNAs survived (Fig. 1B). Finally, the prognostic significance of DE-miRNAs was assessed by biochemical recurrence (BCR) data and overall survival (OS) data of patients in GSE21036 and TCGA databases. Thirteen DE-miRNAs had prognostic value in BCR-free survival (2 upregulated: miR-671-5p, miR-130b-3p; 11 downregulated: miR-221-5p, miR-133b, miR-455-5p, miR-27b-3p, miR-145-3p, miR-23b-3p, miR-1-3p, miR-204-5p, miR-205-5p, miR-133a-3p, miR-222-3p; Table S2), but not in OS survival.

### MiR-671 expression is increased in metastatic PCa tissues, and high expression of miR-671 indicates poor prognosis

The role of majority DE-miRNAs we identified have been investigated; however, the molecular mechanism of miR-671 in PCa metastasis was still unclear. First, MiR-671 expression was evaluated in PCa datasets (GSE21036, TCGA). As shown in Fig. 1C–E, the expression levels of miR-671 increased in PCa tissues compared with ANT. Importantly, the expression levels of miR-671 increased steadily from ANT, PPCa, to MPCa. We also used dbDEMC 2.0^9^ to analyze the expression of miR-671 in other tumors, and found that its expression was upregulated in most tumors (Table S3). This result suggested that miR-671 may be a miRNA with extensive carcinogenic effects. Then, we examined miR-671 expression in our clinical PPCa tissues and ANT, and found that miR-671 expression was elevated in PPCa tissues compared with that in ANT (Fig. 1F, G). We further examined miR-671 expression in normal prostate epithelial cells (RWPE-1) and PCa cells, and found that miR-671 expression was significantly upregulated compared with RWPE-1 (Fig. 1H).

**Fig. 2:**
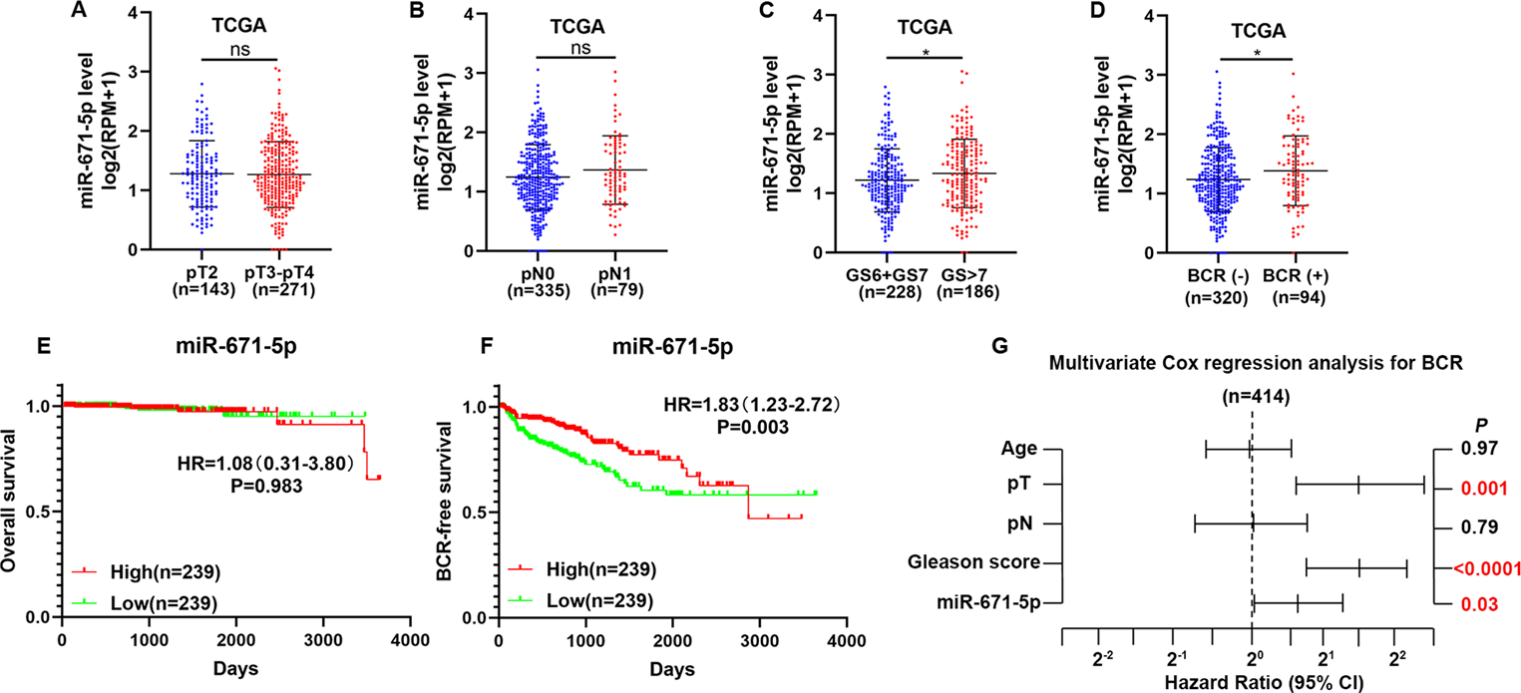
High expression of miR-671 positively correlated with advanced clinicopathological characteristics and indicated poor prognosis. **A**–**D** miR-671 expression levels in PCa tissues with different tumor stage, lymph node metastasis status, Gleason score, and BCR status. **E**, **F** Kaplan–Meier analysis of overall survival curves and BCR-free survival curves of PCa patients with different miR-671 expression levels. The median of miR-671-5p expression was used to stratify the samples. **G** Multivariate Cox-regression analysis for BCR-free survival in TCGA. ^ns^*P* > 0.05; *P < 0.05; Student’s *t*-test. pT, pathologic tumor stage; pN, pathologic lymph node metastasis; GS, Gleason score; BCR, biochemical recurrence; HR, hazard ratio; CI, confidence intervals.

To identify the clinical significance of miR-671 in PCa, we examined the correlation of miR-671 expression with clinicopathological characteristics in PCa patients in GSE21036 and TCGA. The expression of miR-671 was not associated with tumor stage and lymph node metastasis (Fig. 2A, B and Fig. S1A–C). As shown in Fig. 2C, D and Fig. S1D, E, miR-671 expression level positively correlated with Gleason score, and BCR status in PCa patients. High miR-671 expression demonstrated that shorter BCR-free survival, but had no effect on OS in PCa patients (Fig. 2E, F). Univariate and multivariate Cox-regression analysis indicated that miR-671 may be used as independent factors to predict BCR-free survival (HR, 1.61; 95% CI, 1.05-2.45; *P* = 0.03; Fig. 2G and Table S4). The receiver operating characteristic (ROC) curve was also established to evaluate the predictive value of miR-671 expression for distant metastasis status, pathological lymph node metastasis status, and BCR status. The area under the ROC curve (AUC) demonstrated that miR-671 had good predictive value for the metastasis status of PCa patients (AUC: 0.96 in TCGA, 0.85 in GSE21036; Fig. S2 and Table S5). Our results suggested that high expression of miR-671 predicted poor prognosis.

### MiR-671 facilitates PCa cells proliferation, migration, and invasion in vitro and in vivo

**Fig. 3:**
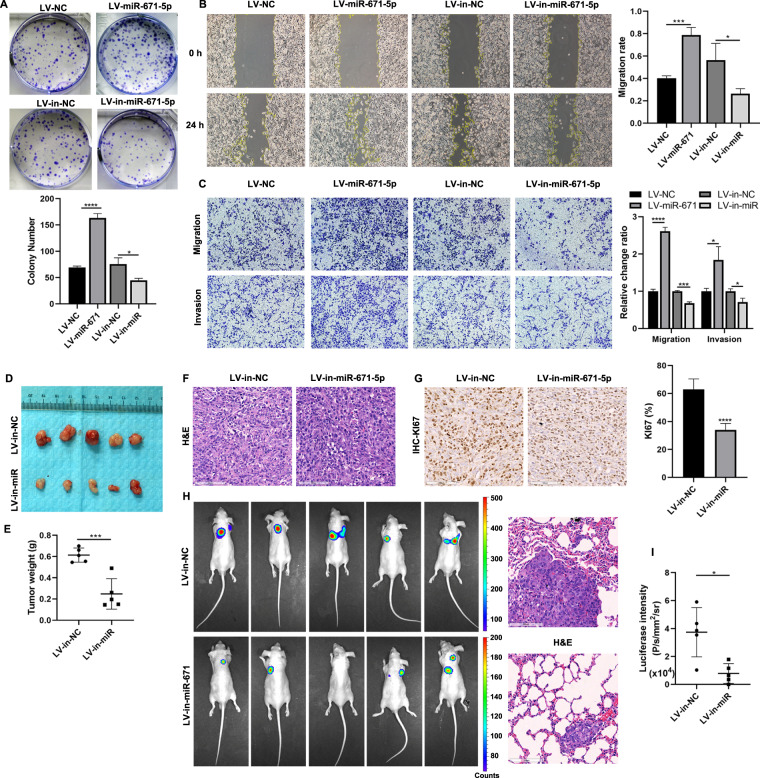
MiR-671 facilitated PCa cells proliferation, migration, and invasion in vitro and in vivo. Colony formation assays (**A**), wound healing assays (**B**), and Transwell assays (**C**) were performed to assess the proliferation, migration, and invasion ability in miR-671 knockdown or overexpressing PC-3 cells. **D** In vivo PCa tumor formation in the xenograft male nude mouse models. **E** Final tumor weights were measured. **F** The xenograft tumor tissues were stained with H&E. **G** KI67 IHC staining in xenografts with LV-in-miR-671 cells or LV-in-NC cells. (**H**-left and **I**) The tail vein xenograft model was used to investigate the effects of miR-671 on PCa metastasis in vivo, and lung colonization ability was measured by bioluminescence imaging. (**H**, right) The lung sections were stained with H&E. Magnification, ×200. Scale bars, 100 μm. The data were presented as means ± SD from three biological replicates. *P < 0.05; **P < 0.01; ***P < 0.001; ****P < 0.0001; Student’s *t*-test.

To detect the function of miR-671 in PCa cell in vitro, we established two PCa cell lines (C4-2, relatively lower expression; PC-3, relatively higher expression) with stable miR-671 overexpression or knockdown by lentivirus infection. Successful overexpression or knockdown of miR-671 was confirmed by qPCR (Fig. S3). Colony formation assays, Annexin/PI staining, wound healing assays, and Transwell assays were performed to assess the role of miR-671. Overexpression of miR-671 significantly promoted proliferation, migration, and invasion of PCa cells (Fig. 3A–C and Fig. S4). Simultaneously, knockdown of miR-671 expression could suppress the ability of proliferation, migration, and invasion in PCa cells. According to the results of Annexin/PI staining (Fig. S5), miR-671 did not participate in the apoptosis process of PCa cells.

To investigate the effect of miR-671 on PCa cell proliferation in vivo, PC-3/LV-in-miR-671 cells or PC-3/LV-NC cells were injected subcutaneously in male nude mice. Knockdown of miR-671 significantly suppressed the tumorigenicity of PC-3 cells in nude mice (Fig. 3D, E). H&E staining showed the histopathological features of the tumor tissues (Fig. 3F). IHC staining demonstrated that KI67 protein, representing cell proliferation ability, decreased in xenografts with LV-in-miR-671 cells (Fig. 3G). These results shown that miR-671 seemed to play an important role in regulating cell proliferation.

To investigate the effects of miR-671 on PCa metastasis in vivo, the tail vein xenograft model was used, where the luciferase-labeled PC-3/LV-in-miR-671 cells and PC-3/LV-NC cells were injected into the tail vein of male nude mice. As shown in Fig. 3H, I, mice injected with LV-in-miR-671 cells exhibited less lung colonization ability compared to the control group with LV-NC cells by bioluminescence imaging, and H&E staining revealed that knockdown of miR-671 significantly reduced the tumor burden in lung. Collectively, our results demonstrated that silencing miR-671 inhibited the metastasis of PCa in vivo.

### NFIA is the direct target of miR-671 to promote PCa progress

**Fig. 4:**
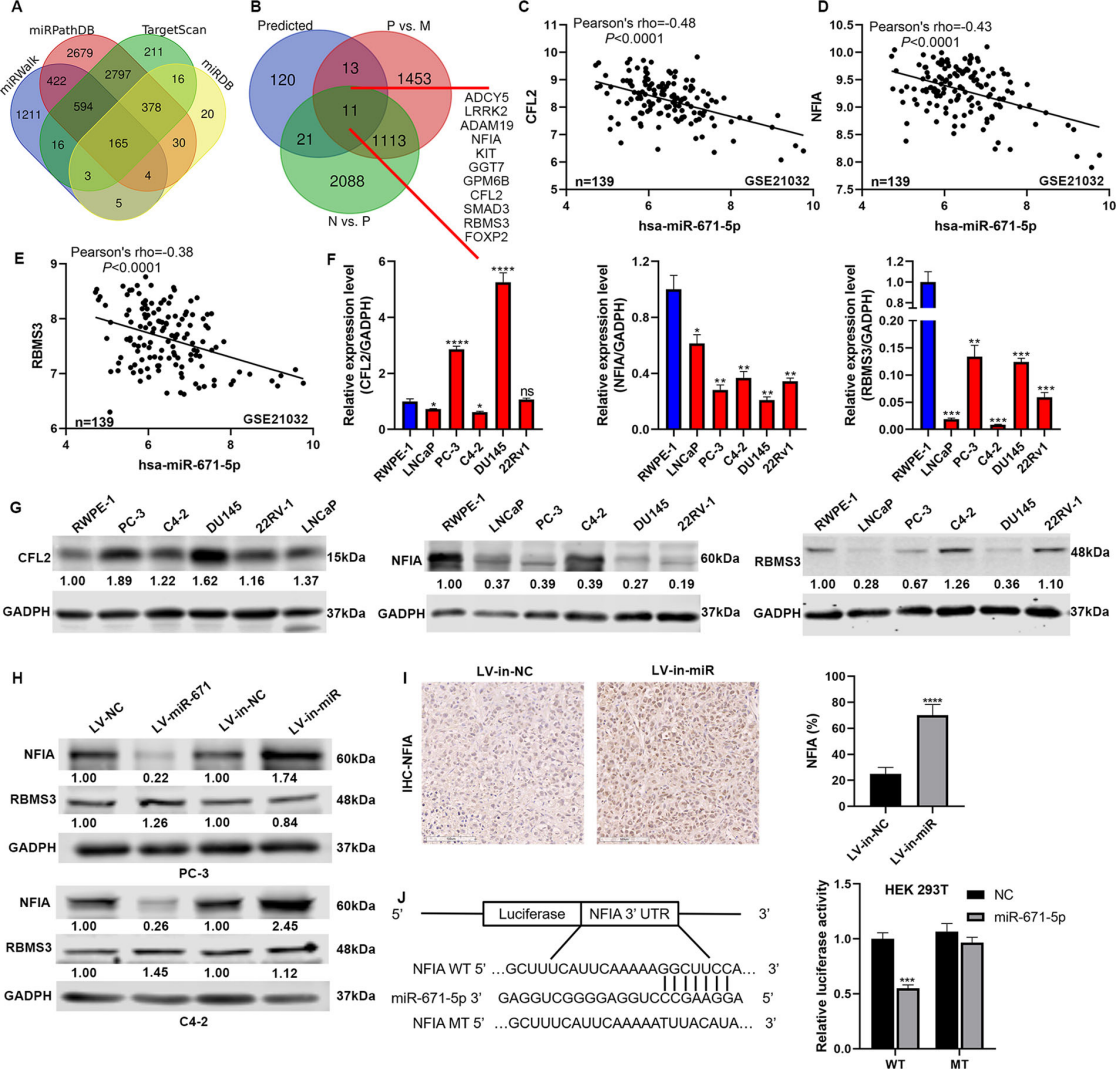
NFIA was the direct target of miR-671. **A**, **B** Identify potential miR-671 target genes by 4 common miRNA prediction databases and GSE21034. **C**–**E** The correlations between miR-671 expression and CFL2, NFIA, and RBMS3 expression. **F**, **G** CFL2, NFIA and RBMS3 mRNA and protein expressions in RWPE-1 and PCa cell lines were examined by qPCR and WB. **H** WB analysis revealed a negatively correlation of NFIA expression with miR-671 expression in PCa cells. RBMS3 did not accept the regulation of miR-671. **I** NFIA IHC staining in xenografts with LV-in-miR-671 cells or LV-in-NC cells. **J**-Left: potential miR-671 binding sequences in the 3’UTR of NFIA mRNAs. **J**-Right: luciferase activity assays showed that the overexpression of miR-671 significantly reduced the luciferase activity of binding site of NFIA, and the mutation of binding site blocked the interaction. Magnification, ×200. Scale bars, 100 μm. The data were presented as means ± SD from three biological replicates. *P < 0.05; **P < 0.01; ***P < 0.001; ****P < 0.0001; Student’s *t*-test. N, adjacent normal tissues; P, primary localized PCa tissues; M, metastatic PCa tissues; WT, wide type; MT, mutant type.

To identify how miR-671 was involved in PCa progress, we used 4 miRNA target prediction databases, Target Scan^10^, miRDB^11^, miRPathDB^12^, and miRWalk^13^, to identify the target genes of miR-671. According to the analysis, 165 candidate genes were identified as targets of miR-671 (Fig. 4A). Considering the expression characteristics of miR-671 in PCa, we also identified 1124 differentially expressed genes (DEGs) which expression decreased steadily from ANT, PPCa, to MPCa using GSE21034, and 11 candidate genes belonged to them (Fig. 4B). Importantly, miR-671 had significant negative correlations with CFL2 (Pearson’s rho = −0.48), NFIA (Pearson’s rho = −0.43) and RBMS3 (Pearson’s rho = −0.38) (Fig. 4C–E).

To investigate whether CFL2, NFIA, and RBMS3 contributed to the development of PCa, we first examined their expression by qPCR and WB in PCa cells (Fig. 4F, G). The mRNA and protein level of RBMS3 and NFIA declined in PCa cells compared with that in RWPE-1. These results were in accordance with the outcomes analyzed from GSE21034. Then, we investigated whether NFIA and RBMS3 expression were regulated by miR-671. WB and qPCR were performed to detect NFIA and RBMS3 in PCa cells with stable overexpression or knockdown of miR-671 (Fig. 4H and Fig. S6). PCa cells with stable overexpression of miR-671 showed a significant decrease in NFIA protein expression. In contrast, the down-regulation of miR-671 significantly increased the NFIA protein expression. RBMS3 did not accept the regulation of miR-671. IHC staining revealed that NFIA protein was elevated in xenografts with LV-in-miR-671 cells (Fig. 4I). According to the bioinformatics analysis, NFIA had the binding site for miR-671 in 3’UTR region (Fig. 4J, left). So, we performed luciferase activity assays to determine whether miR-671 directly interacted with the 3’UTR of NFIA mRNA. The overexpression of miR-671 significantly reduced the luciferase activity of binding site of NFIA, and the mutation of binding site blocked the interaction (Fig. 4J, right).

**Fig. 5:**
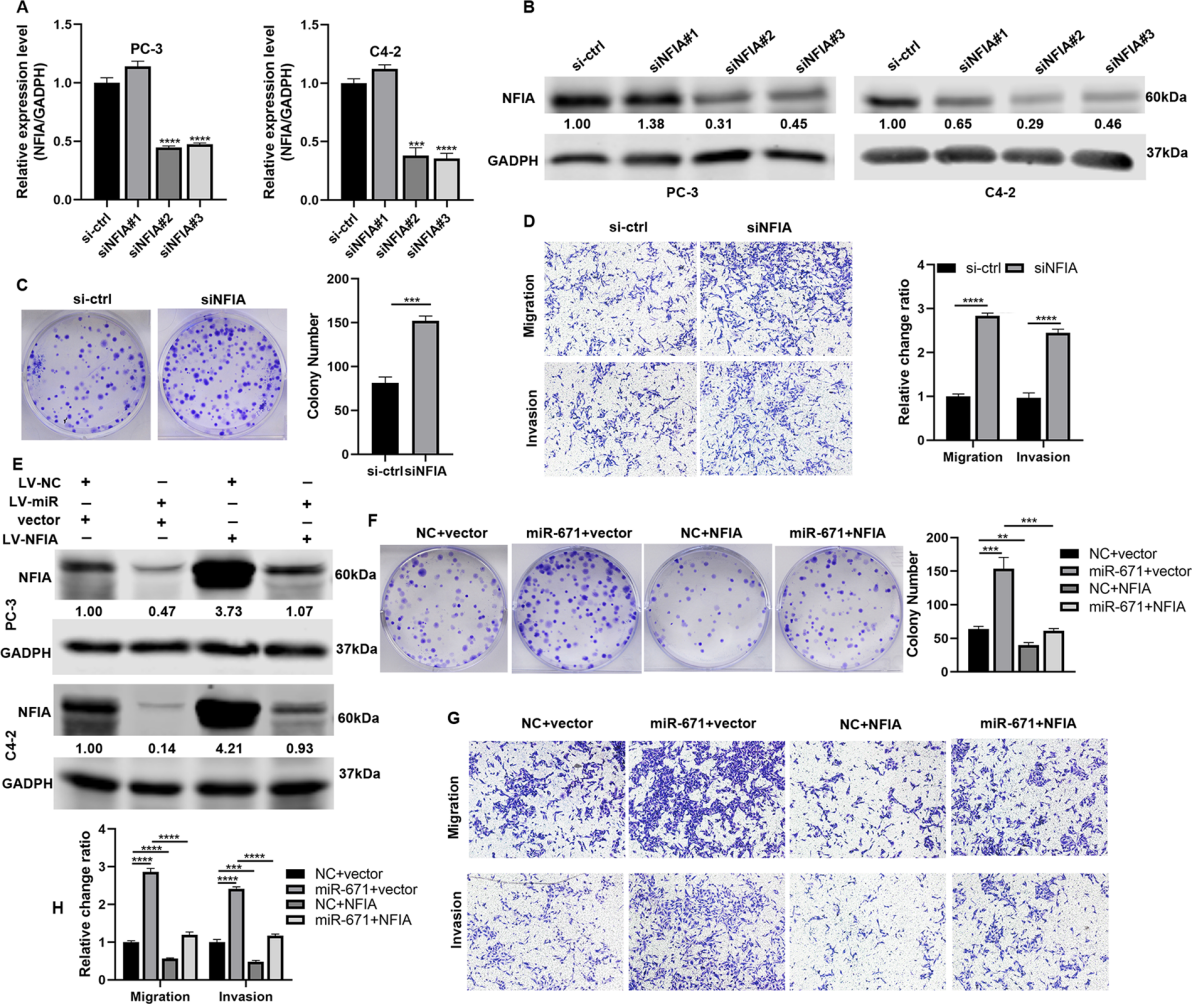
MiR-671 promoted tumor development and metastasis via downregulating of NFIA. **A**, **B** Three siRNAs were designed to suppress NFIA expression, and si-NFIA #2 was chosen for the subsequent experiments due to the highest inhibitory efficiency. **C**, **D** Promotive effect on proliferation, migration, and invasion in PC-3 cells after Knockdown of NFIA expression. **E** NFIA protein expression in rescue experiment of NFIA overexpression in cells with stable miR-671 overexpression and corresponding control cells. **F**–**H** The proliferative, migratory, and invasive abilities of PC-3 cells impaired after upregulating NFIA, and the carcinogenesis effect of miR-671 could also be reversed. The data were presented as means ± SD from three biological replicates. ^**^P < 0.01; ^***^P < 0.001; ^****^P < 0.0001; Student’s *t*-test.

To verify whether silencing NFIA was essential for PCa proliferation and metastasis, 3 siRNAs targeting NFIA were designed. Si-NFIA #2 was chosen for the subsequent experiments due to the highest inhibitory efficiency (Fig. 5A, B). Knockdown of NFIA expression could facilitate the ability of proliferation, migration, and invasion in PCa cells (Fig. 5C, D and Fig. S7A, B).

To further confirm whether miR-671 promoted PCa development and metastasis through NFIA, we performed rescue experiment of NFIA overexpression in cells with stable miR-671 overexpression and corresponding control cells (Fig. 5E). As shown in Fig. 5F–H, Fig. S7C, D, the proliferative, migratory, and invasive abilities of PCa cells impaired after upregulating NFIA, and the carcinogenesis effect of miR-671 could also be reversed. Our results suggested that NFIA was the direct target of miR-671 to promote PCa progress.

### MiR-671-5p regulates CRYAB expression through the intermediary NFIA

**Fig. 6:**
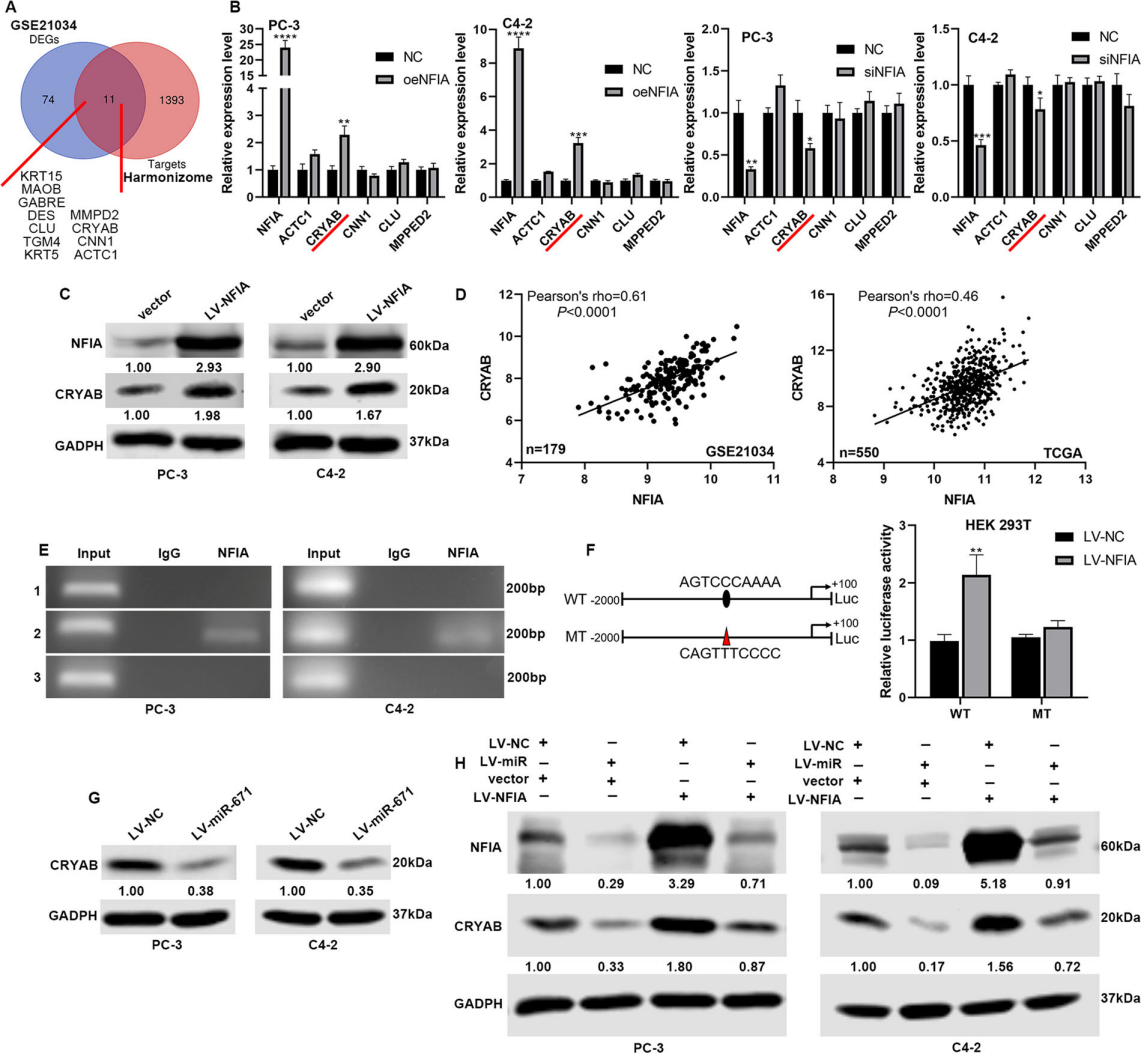
MiR-671 regulated CRYAB expression through NFIA. **A** Bioinformatics prediction of potential NFIA targets by GSE21034 and Harmonizome tool, and 11 genes were identified. **B** Expression of 5 target genes were measured by qPCR in PC-3 and C4-2 cells. **C** Expression of CRYAB protein were measured by WB in PC-3 and C4-2 cells. **D** The correlations between NFIA expression and CRYAB expression in GSE21034 and TCGA datasets. **E** ChIP assay confirmed the direct binding of NFIA on the CRYAB promoter. **F** Luciferase activity assays shown that the overexpression of NFIA significantly promoted the luciferase activity of binding site of CRYAB, and the mutation of binding site blocked the interaction. **G** The results of WB demonstrated that miR-671 could negatively regulate the expression of CRYAB. **H** WB analysis was performed to detect the expression changes of NFIA and CRYAB proteins after infecting PCa cells with LV-miR-671 and LV-NFIA simultaneously. The data were presented as means ± SD from three biological replicates. **P < 0.01; ***P < 0.001; ****P < 0.0001; Student’s *t*-test.

NFIA, as a transcription factor (TF), could combine with the promoter region of downstream target genes to regulate their transcription process. Therefore, we predicted the target genes of NFIA through the Harmonizome tool^14^. According to the analysis, we identified 1404 candidate genes as targets of NFIA (Fig. 6A). We also identified 85 DEGs between ANT and tumor using GSE21034 (adj. *P* value < 0.05, |Fold change | > 2), and 11 candidate genes belonged to them (Fig. 6A). The web tool, cBioPortal, were performed to identify the relationship between NFIA expression and target genes expression in PCa (Table S6). Interestingly, these target genes all positively correlated with NFIA, except TGM4. Then, we selected 5 genes, associated with tumor development and metastasis in PubMed, for further verification. The results of qPCR and WB confirmed that NFIA could positively regulate the expression of CRYAB (Fig. 6B, C). NFIA had significant positive correlation with CRYAB in PCa (Pearson’s rho: 0.61 in GSE21034, 0.46 in TCGA; Fig. 6D).

To further explore the underlying mechanism between NFIA and CRYAB, we firstly used the JASPAR database^15^ to predict the NFIA binding sites within the CRYAB promoter. According to the analysis, 3 binding sites were identified: #1—TGTGCCATGT; #2—AGTCCCAAAA; #3—AGTTGCCACA (5’–3’). ChIP assays were performed to determine whether NFIA could bind directly to the CRYAB promoter. As shown in Fig. 6E, the results showed that NFIA directly bound to the CRYAB promoter (site #2) and not to negative control. Then, luciferase activity assays were performed to further confirm this finding. We constructed the pGL3-CRYAB-WT and pGL3-CRYAB-MT luciferase reporters respectively (Fig. 6F, left). The overexpression of NFIA significantly promoted the luciferase activity of binding site of CRYAB, and the mutation of binding site blocked the interaction (Fig. 6F, right). Our results demonstrated that NFIA played the biological function by upregulating CRYAB.

Considering the above findings, could miR-671 regulate CRYAB expression through NFIA? Firstly, we confirmed the relationship between miR-671 and CRYAB by bioinformatics analysis, qPCR, WB, and IHC of mice xenografts (Fig. 6G and Fig. S8A–C). The results revealed that miR-671 could negatively regulate the expression of CRYAB. According to the in-silico analysis, CRYAB had no binding sites for miR-671 in 3’ UTR region. Therefore, miR-671-5p could not directly regulate the expression of CRYAB, and this regulatory relationship required the existence of an intermediary. Then, we infected PCa cells with LV-miR-671 and LV-NFIA simultaneously. Western blot results showed that upregulation of NFIA in PCa cells with miR-671 overexpression restored CRYAB expression (Fig. 6H). These results confirmed that miR-671 regulated CRYAB expression through NFIA.

In last, we performed rescue experiment of CRYAB overexpression in cells with stable miR-671 overexpression and corresponding control cells (Fig. S9A). As shown in Fig. S9B, C, the proliferative, migratory, and invasive abilities of PCa cells impaired after upregulating CRYAB, and the carcinogenesis effect of miR-671 can also be reversed. Our results demonstrated that NFIA/CRYAB axis contributed to miR-671-associated PCa development and metastasis.

### Suppressed NFIA/CRYAB axis promotes PCa progress and is associated with poor prognosis

To identify the clinical significance of NFIA and CRYAB in PCa, we firstly examined the correlation of their expression levels with clinicopathological characteristics in PCa patients in GSE21034. The expression levels of NFIA and CRYAB decreased steadily from ANT, PPCa, to MPCa in GSE21034 (Figs. S10A, S11A). Their expression negatively correlated with advanced clinicopathological characteristics (pathological tumor stage, pathological lymph node metastasis, Gleason score, and BCR status) in PCa patients (Figs. S10C–F, S11C–F). Low NFIA or CRYAB expression demonstrated that shorter BCR-free survival, but had no effect on OS in PCa patients (Figs. S10G, H, S11G, H). Univariate and multivariate Cox-regression analysis indicated that NFIA and CRYAB could predict BCR-free survival in PCa patients, and CRYAB may be used as independent factors to predict BCR-free survival (Table S7 and S8). The same analysis was also performed in TCGA dataset, and the results were similar to those obtained in GSE21034 (Fig. S12, Tables S9 and S10).

**Fig. 7:**
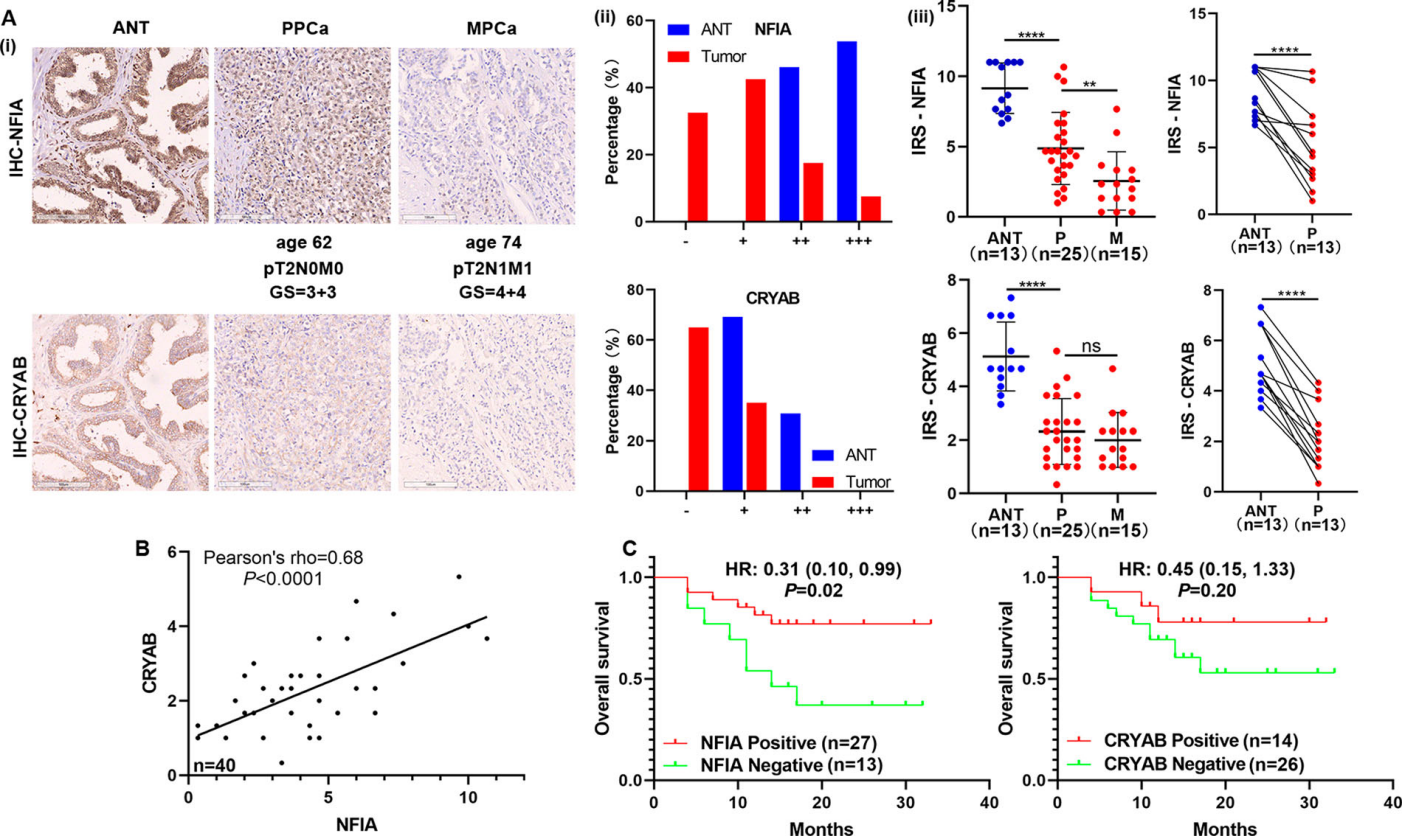
Low NFIA and CRYAB expressions were associated with metastasis and poor prognosis in PCa. **A** NFIA and CRYAB expressions in 40 PCa tissue samples were detected by IHC. NFIA and CRYAB were spatially correlated. Representative IHC photographs of adjacent normal tissues (ANT) and tumor tissue samples (PPCa, MPCa) were shown as indicated. (i) The proportion of PCa tissues and adjacent normal tissues with negative (−), weak (+), moderate (++) and strong (+++) NFIA or CRYAB staining intensity. (ii) NFIA and CRYAB protein expressions in PCa tissues and adjacent normal tissues were quantified, and their expression were significantly decreased in PCa tissues with metastasis (iii). **B** NFIA and CRYAB expression levels in PCa tissue were positively correlated. **C** Kaplan–Meier analysis of overall survival curves of PCa patients with different NFIA or CRYAB expression levels. Magnification, ×200. Scale bars, 100 μm. The data were presented as means ± SD. ^ns^*P* > 0.05; ^**^P < 0.01; ^****^P < 0.0001; Student’s *t*-test. ANT, adjacent normal tissues; P or PPCa, primary localized PCa tissues; M or MPCa, metastatic PCa tissues; HR, hazard ratio.

Then, we verified the protein expression of NFIA and CRYAB in the HPA database (https://www.proteinatlas.org/). According to the IHC analyses of NFIA protein, NFIA showed higher expression in ANT than in PCa tissues (expression level: ANT—3/3, moderate. Tumor—3/12, moderate; 7/12, low; 2/12, negative) (Fig. S13). But the expression of CRYAB protein could not be queried in the HPA database. Furthermore, IHC of 13 ANT tissues, 25 PPCa tissues and 15 MPCa tissues showed that the expression of both NFIA and CRYAB was dramatically decreased in PCa samples, especially in MPCa tissues (Fig. 7A). And our results showed a protein expression correlation between NFIA and CRYAB (Pearson’s rho = 0.68, *P* < 0.0001, Fig. 7B). Furthermore, PCa patients with NFIA negative had a lower OS than NFIA positive patients (HR, 0.31; 95% CI, 0.10-0.99; *P* = 0.02; Fig. 7C). The OS of PCa patients with CRYAB negative declined, compared to that of CRYAB positive patients, but the difference was not statistically significant (*P* = 0.20, Fig. 7D). We then analyzed the correlation of these two proteins with clinicopathological characteristics in PCa patients. Chi-square analysis revealed that NFIA level was significantly associated with tumor metastasis (*P* = 0.004, Table 1). Our results demonstrated that the suppressed NFIA/CRYAB axis promoted PCa progress and was associated with poor prognosis.

**Table 1:** Correlation between NFIA and CRYAB expression with clinicopathological characteristics in PCa (*n* = 40).

		NFIA			CRYAB		
	*n*	Positive (%)	Negative (%)	*P*^a^	Positive (%)	Negative (%)	*P*^a^
*Age (yeas)*^b^							
≤72	23	17 (73.9)	6 (26.1)	0.31	9 (39.1)	14 (60.9)	0.52
>72	17	10 (58.8)	7 (41.2)		5 (29.4)	12 (70.6)	
*pT*							
T2	26	18 (69.2)	8 (30.8)	0.75	9 (34.6)	17 (65.4)	0.95
T3–T4	14	9 (64.3)	5 (35.7)		5 (35.7)	9 (64.3)	
* GS*							
<7	5	3 (60.0)	2 (40.0)	0.70	4 (80.0)	1 (20)	0.10
≥7	35	24 (68.6)	11 (31.4)		10 (40)	15 (60)	
* pN*							
N0	28	20 (71.4)	8 (28.6)	0.42	12 (42.9)	16 (57.1)	0.11
N1	12	7 (58.3)	5 (41.7)		2 (16.7)	10 (83.3)	
* M*							
M0	25	21 (84.0)	4 (16.0)	0.004	10 (40.0)	15 (60.0)	0.40
M1	15	6 (40.0)	9 (60.0)	\	4 (26.7)	11 (73.3)	

NFIA and CRYAB expression was determined by IHC.

*pT* pathologic tumor stage, *GS* gleason score, *pN* pathologic lymph node metastasis, *M* distant metastasis.

^a^Chi-square test.

^b^mean age.

## Discussion

PCa is the second most common cancer among men worldwide. Due to the uneven distribution of medical resources, prostate-specific antigen screening has not been carried out in many areas of China. A large number of patients are already in the advanced stage of the disease at the time of initial diagnosis, and more than half of patients are diagnosed with PCa with distant metastasis^16^. Metastasis is the leading cause of poor prognosis and mortality in PCa patients. Hence, identifying underlying molecular mechanisms in PCa metastasis is important for improving diagnostic and therapeutic strategy of PCa.

Though functions of miRNAs in cancer have been extensively studied, there are still many other miRNAs that play important regulatory roles in metastasis that have not yet been discovered. In this study, we identified 13 DE-miRNAs, which may play a key regulatory role in the metastasis of PCa, by bioinformatics analysis. Two upregulated miRNAs had worse BCR-free survival, and 11 downregulated miRNAs had better BCR-free survival. For 11 downregulated miRNAs, studies have described their molecular mechanism in PCa, and their researches were consistent with our findings. These downregulated miRNAs repressed tumor development and metastasis in PCa^17–27^. This proved the reliability of our screening results from the side. The roles of miR-130b-3p in PCa were controversial. Some studies have reported that miR-130b-3p was an oncogene in PCa^28,29^. However, other researches revealed that miR-130b-3p inhibited PCa proliferation, angiogenesis, and metastasis^30–32^. The role of miR-671-5p (miR-671) was tissue specific. MiR-671 played the role of oncogene in clear cell renal cell carcinoma^33^, glioma^34^ and colon cancer^35^ and the role of tumor suppressor in breast cancer^36^, esophageal squamous cell carcinoma^37^, osteosarcoma^38^, and gastric cancer^39^. In PCa, only one research reported that miR-671 facilitated PC-3 cell proliferation in vitro^40^, though the function of miR-671 in cancer metastasis and in vivo was still unknown. Our study demonstrated that miR-671 was upregulated in PCa, especially in PCa with metastasis, and promoted PCa development and metastasis in vitro and vivo via NFIA/CRYAB axis.

In this study, we first evaluated the expression of miR-671 and its clinical value in PCa. The results revealed that miR-671 was associated with advanced clinicopathological characteristics, and its high expression predicted poor prognosis. Thereafter, using a series of in vitro and in vivo assays, we found that miR-671 acted as a development and metastasis accelerator in PCa. Ours was the first research that provided the comprehensive evaluation of the role of miR-671 in PCa.

Studies have been reported that miR-671 played the role of oncogene or tumor suppressor by targeting TRIM67, APC, CDR1, SOX6, URGCP, CD44, DDX5, and FGFR2^33–40^. NFIA, a transcription factor, was identified as the direct target of miR-671 in our study. NFIA, a member of nuclear factor I (NFI) family, functioned as an oncogene in esophageal carcinoma^41^ and glioma^42^. NFIA also played a tumor suppressor role in tumors. For example, NFIA could restore radiosensitivity by downregulating the AKT and ERK pathways in non-small cell lung cancer^43^. In PCa, Grabowska et al.^44^ demonstrated that NFI family members could regulate prostate-specific gene expression by interacting with FOXA1. We confirmed for the first time that NFIA, regulated by miR-671, could inhibit PCa cells proliferation, migration and invasion, and function as a tumor suppressor in PCa.

Furthermore, we found NFIA positively regulated the transcription of CRYAB. CRYAB, a member of small heat shock protein family, was first discovered in the lens of eye^45^. Studies reported that the expression of CRYAB increased in 10/13 tumors and decreased specifically in 3/13 tumors (PCa, anaplastic thyroid carcinoma, ovarian cancer)^46^. CRYAB may be regulated by Ets1 (breast cancer)^47^, KLF4 (osteosarcoma)^48^, HSFI (hepatocellular carcinoma)^49^. Valcarcel-Jimenez et al.^50^ found MITF exerted tumor-suppressive activity in PCa, and CRYAB was the direct target of MITF. However, there was a complex regulatory network between molecules. In our study, we found NFIA could directly bind to the promoter region of CRYAB, and CRYAB mediated the tumor-suppressive activity of NFIA in PCa. Importantly, miR-671 could regulate CRYAB expression through the intermediary NFIA. In addition, the expression of NFIA and CRYAB decreased steadily from ANT, PPCa, to MPCa. Their expression level negatively correlated with advanced clinicopathological characteristics, and low NFIA or CRYAB expression demonstrated that shorter BCR-free survival and OS.

In conclusion, our results demonstrated that miR-671-5p promoted PCa development and metastasis by suppressing NFIA/CRYAB axis. Clearly clarifying the miR-671-5p/NFIA/CRYAB axis could facilitate the development of diagnostic and therapeutic strategy of PCa.

## Materials and methods

### Human tissues and cell lines

Total of 53 prostate samples, in which included adjacent ANT (*n* = 13), PPCa tissues (*n* = 25), and MPCa tissues (*n* = 15), were collected from January 2013 to December 2018 in our Institute. All tissue types were confirmed through hematoxylin and eosin (H&E) staining by 2 pathologists. This study was approved by the Ethics Committee of the First Affiliated Hospital for Guangzhou Medical University, and all patients have signed informed consents.

The normal prostate epithelial cell line RWPE-1 and LNCaP PCa cell line were purchased from Chinese Academy of Sciences cell bank (Shanghai, China). PC-3M, 22RV-1, and C4-2 PCa cell lines were stored in our laboratory. PC-3 and DU145 PCa cell lines were presented by K. Weiting from Shandong University. Cell lines were authenticated by STR profiling, tested negative for mycoplasma. All cells were cultured as described previously^51^, and grew in 5% CO_2_ at 37 °C.

### TCGA and GEO database analysis

The GSE21032 datasets were downloaded from the GEO database (https://www.ncbi.nlm.nih.gov/geo/), and the TCGA prostate adenocarcinoma (PRAD) dataset was downloaded from the UCSC Xena database platform (http://xena.ucsc.edu/). GSE21032 was an integrated genome analysis dataset of human prostate cancer, which included two subsets: GSE21036 (miRNA sequencing data) and GSE21034 (mRNA sequencing data). Table S11 shown the basic information of included datasets. These datasets all have related clinical date and follow-up data. We used these data to analyze gene expression and the correlation between gene expression and the clinicopathological characteristics and metastasis status of PCa patients. When conducting different analyses, the principles of sample selection were: (1) when analyzing gene expression or correlation between mRNA and miRNA, we used all samples with gene expression information; (2) when performing the Kaplan–Meier curves, we used all samples with gene expression information and follow-up information (BCR, OS); (3) when analyzing the correlation of gene expression with clinicopathological characteristics of PCa patients and performing univariate and multivariate Cox-regression analysis, we used samples with complete clinical pathological parameters (tumor stage status, distant metastasis status, pathological lymph node metastasis status, and BCR status) and gene expression information.

### Small interfering RNA, transfection, and generation of stable cell lines

Three small interfering RNAs (siRNAs) targeting NFIA (Gene Pharma, Jiangsu, China) were used to knockdown endogenous NFIA: siNFIA-1, 5′-CCAGGAUGAAUUUCAUCCUTT-3′; siNFIA-2, 5′-GAAGGAUGAAUUGCUAAGUTT-3′; siNFIA-3, 5′-GGAGGUUGGACCUUGUUAUTT-3′. Cells were transfected with Lipofectamine 3000 (Invitrogen, Carlsbad, CA, USA) according to the manufacturer’s protocol.

Stable cell lines expressing miR-671, in-miR-671, NFIA, and CRYAB were generated using the lentivirus (Gene Pharma, Jiangsu, China). Stable cell lines were selected for 10 days with Puromycin (PC-3, 2 μg/mL; C4-2, 4 μg/mL) or G418 (500 μg/mL).

### Mice xenograft and tumor metastasis

All procedures related to the experimental animals were authorized by the Animal Care and Use Committee of the First Affiliated Hospital of Guangzhou Medical University. Twenty 5 weeks-old male BALB/c nude mice were obtained from the Experimental Animal Center of Guangdong Province (Guangzhou, China) and raised under Specific Pathogen Free (SPF) conditions. Stable PC-3 cells (2 × 10^6^) infected with LV-in-miR-671/LV-in-NC and LV-luciferase were injected subcutaneously for tumorigenesis assay (*n* = 5/group, randomly assigned). After 7 weeks, the mice were sacrificed by cervical dislocation, and the tumors were dissected and weighed. To assess cells metastasis ability, 4 × 10^6^ cells in 100 μL PBS were injected into the tail veins of mice (*n* = 5/group, randomly assigned). After 7 weeks, tumor cells metastasis in nude mice was detected using a small animal live imaging system (PerkinElmer, Waltham, MA, USA). Then, the mice were sacrificed, and the lung was dissected. Mice xenografts and lung tissues were embedded in paraffin wax for H&E staining and immunohistochemical (IHC) assay using antibodies against NFIA (Abcam, Shanghai, China, #ab228897), CRYAB (Proteintech, Wuhan, Hubei, China, #15808-1-AP), and KI67 (Proteintech, #27309-1-AP). Researchers have always been aware of the grouping of animal experiments.

### Dual-luciferase reporter assays

HK293T were plated in 96-well plates and transfected with 100 ng of pGL3-NFIA-WT or pGL3-NFIA-MT (pGL3-CRYAB-WT or pGL3-CRYAB-MT) luciferase plasmids by lipofectamine 3000 (Invitrogen, Carlsbad, CA, USA). After 48 h, Luciferase and Renilla signals were measured using Dual-Luciferase Reporter Assay System (Promega, Madison, WI, USA) according to the manufacturer’s protocol.

### Chromatin immunoprecipitation (ChIP)

We predicted 3 binding sites of NFIA and CRYAB promoter regions: Site 1. TGTGCCATGT; Site 2. AGTCCCAAAA; Site 3. AGTTGCCACA. ChIP was conducted as previously described^52^. Chromatin solutions were immunoprecipitated with a specific anti-NFIA antibody (Abcam, Shanghai, China, #ab228897) and normal Rabbit IgG antibody (Cell Signaling Technology, #2729) for control. The primers used for PCR amplification can be found in Table S12. The PCR amplified products were subjected to agarose gel electrophoresis, and the results were analyzed using the gel imaging analyzer (Syngene, Cambridge, UK).

Other conventional experimental operation methods, such as Q-PCR, WB, IHC, Colony formation assays, Migration and invasion assays, Annexin/PI staining, can be found in Supplementary materials.

### Statistical analysis

All the statistical analyses were conducted using SPSS version 20.0 (SPSS Inc., Chicago, IL, USA) and GRAPHPAD PRISM version 8.0 (GraphPad Software, San Diego, CA, USA). Data were expressed as the mean ± SD. Student’s *t*-test, paired *t*-test, and Chi-square test were performed to determine statistical differences between two groups. MiRNAs and mRNAs expression were explored by Pearson’ or Spearman’s correlation. Kaplan–Meier survival curves were plotted. The biochemical recurrence (BCR) free survival and overall survival (OS) were assessment by hazard ratio (HR), 95% confidence intervals (CI), and log rank *P*-value. The data meet the assumptions of the tests. *P* < 0.05 indicated statistical significance.

## Supplementary information

Supplementary materials and method

Supplementary Figure and Table Legends

Figure S1

Figure S2

Figure S3

Figure S4

Figure S5

Figure S6

Figure S7

Figure S8

Figure S9

Figure S10

Figure S11

Figure S12

Figure S13

Table S1

Table S2

Table S3

Table S4

Table S5

Table S6

Table S7

Table S8

Table S9

Table S10

Table S11

Table S12
