# Supplementary material for: MiRNA-671-5p Promotes prostate cancer development and metastasis by targeting NFIA/CRYAB axis
Source: Cell Death Dis. 2020 Nov 3;11(11):949. doi: 10.1038/s41419-020-03138-w (PMC7642259; doi:10.1038/s41419-020-03138-w)
Supplement: Supplementary file 1 — Supplementary materials and method [file 41419_2020_3138_MOESM1_ESM.docx]

**Supplementary materials and methods**

**RNA extraction, reverse transcription and quantitative real-time PCR**

Total RNA from prostate tissues and cells was isolated with TRIzol Reagent (Invitrogen; Thermo Fisher Scientific, Inc., Carlsbad, CA, USA) following manufacturer’s instructions. The cDNA of miRNA was synthesized with All-in-One miRNA First-Strand cDNA Synthesis Kit (FulenGen Co., Ltd., Guangzhou, China), and quantitative real-time PCR (qPCR) was conducted using All-in-One miRNA qPCR Kit (FulenGen Co., Ltd., Guangzhou, China). The cDNA of mRNA was synthesized with PrimeScript RT reagent Kit with gDNA Eraser (Takara), and qPCR was conducted using the SYBR green Premix Ex Taq II (Takara). Q-PCR was performed with Applied Bio-systems 7500 Fast Real-Time RCR System (Applied Biosystems, Foster City, CA, USA). Each measurement was performed in triplicate, and the results were normalized to the internal control of U6 or GAPDH. Relative expression of miRNA or mRNA was determined by 2^-△△Ct^ method. Primer information used in the study can be found in Table S12.

**Colony formation assays**

PCa cells (500 cells/well) were placed in 6-well plate and incubated in media containing 10% FBS for 14 days to allow colony formation. Then, colonies were fixed with 4% paraformaldehyde and stained with 0.1% crystal violet. The results were recorded with a camera, and colonies larger than 50 cells were counted.

**Annexin/PI staining**

PC-3 and C4-2 cells were transfected by mimic/NC or inhibitor/NC of miR-671 (Ribobio, Guangzhou, China) by Lipofectamine 3000 (Invitrogen; Thermo Fisher Scientific, Inc., Carlsbad, CA, USA) according to the manufacturer’s protocol. After 48h, cells were harvested to detect cell apoptosis, using Annexin V-FITC/PI staining kit (Key Gen Biotech, Jiangsu, China). Cells (5-10 × 10^4^) were collected and incubated in 500 μL 1X Annexin V buffer. Then, 5 μL Annexin V-FITC solution and 5 μL propidium iodide staining solution was added (mix gently). Cells were incubate for 15 minutes in the dark at room temperature (20-25℃). Cytofluorimetric analysis was performed by the flow cytometer (Millipore, MA, USA).

**Migration and invasion assays**

Wound healing assays were used to verify the migration ability of PCa cells at the 2D level. Cell migration and invasion at 3D level were evaluated using a Transwell permeable support chamber (Corning Incorporated, Corning, NY, USA) with or without Matrigel (BD Biosciences, Franklin Lakes, NJ, USA). All experiments were carried out according to the manufacturer’s instructions.

**Western blot analysis**

Cells were lysed in RIPA Lysis Buffer (ComWin Biotech Co., Ltd., Beijing, China) containing PMSF (1%). Western blot was conducted as previously described^1^. The total proteins were separated by SDS–PAGE and detected with the primary antibodies against NFIA (Proteintech, #11750-1-AP), CRYAB (Proteintech, #15808-1-AP), and RBMS3 (Abcam, #ab181082), respectively. GADPH (SAB, Nanjing, Jiangsu, #21612) was used as internal control. Then, all membranes were incubated with secondary antibody which was anti-rabbit IgG conjugated with horseradish peroxidase (LI-COR, Lincoln, NE, USA, #C91030-13). Infrared imaging system (LI-COR, Lincoln, NE, USA) and Image J software were used to visualize and quantify protein bands.

**Histological and immunohistochemical assessment**

Histological and IHC analysis and evaluation were performed in mice xenografts and lung tissues and clinical PCa samples. H&E and IHC were employed according to a standard method, as described previously^2,3^. PathScope digital scanner (Gene Tech, Shanghai, China) were used to digitize slides, and NFIA and CRYAB expression were quantified according to the protocol described previously respectively^2^.

**Predicting the binding site of NFIA and CRYAB promoter**

The steps to predict the binding site were：1. search for the promoter sequence (2000-3000bp) of CRYAB gene in PubMed; 2. enter the transcription factor name, NFIA, in JASPAR; 3. choose specie as vertebrata; 4. paste the promoter sequence of CRYAB into the input box according to the instructions; 5. click the scan button to get the prediction results; 6. according to the score, select several binding sites for verification.

**References**

1 Sun, X. *et al.* miR-652 Promotes Tumor Proliferation and Metastasis by Targeting RORA in Endometrial Cancer. *Molecular cancer research : MCR* **16**, 1927-1939, doi:10.1158/1541-7786.mcr-18-0267 (2018).

2 Li, E. *et al.* PSCA promotes prostate cancer proliferation and cell-cycle progression by up-regulating c-Myc. *The Prostate* **77**, 1563-1572, doi:10.1002/pros.23432 (2017).

3 Zhao, Z. *et al.* A PSCA/PGRN-NF-kappaB-Integrin-alpha4 Axis Promotes Prostate Cancer Cell Adhesion to Bone Marrow Endothelium and Enhances Metastatic Potential. *Molecular cancer research : MCR* **18**, 501-513, doi:10.1158/1541-7786.mcr-19-0278 (2020).
