# Supplementary material for: MiRNA-671-5p Promotes prostate cancer development and metastasis by targeting NFIA/CRYAB axis
Source: Cell Death Dis. 2020 Nov 3;11(11):949. doi: 10.1038/s41419-020-03138-w (PMC7642259; doi:10.1038/s41419-020-03138-w)
Supplement: Supplementary file 2 — Supplementary Figure and Table Legends [file 41419_2020_3138_MOESM2_ESM.docx]

**Supplementary Figure Legends**

**Figure S1.** High expression of miR-671 positively correlated with advanced clinicopathological characteristics in GSE21036.

(A-E) miR-671 expression levels in PCa tissues with different tumor stage, lymph node metastasis status, Gleason score and BCR status. (F-G) Kaplan–Meier analysis of overall survival curves and BCR-free survival curves of PCa patients with different miR-671 expression levels. The median of miR-671-5p expression was used to stratify the samples. ^ns^*P* > 0.05; ^*^*P* < 0.05; Student’s *t*-test. cT, clinical tumor stage; pT, pathologic tumor stage; pN, pathologic lymph node metastasis; GS, Gleason score; BCR, biochemical recurrence; HR, hazard ratio.

**Figure S2.** ROC curves of miR-671 expression levels for distant metastasis status, lymph node metastasis status and BCR status.

The ROC curve was established to evaluate the predictive value of miR-671 expression for distant metastasis status, lymph node metastasis status, and BCR status in TCGA (A-C) and GSE21036(D-F). We used samples (only tumor samples) with information about distant metastasis status, pathological lymph node metastasis status, and BCR status for corresponding analysis. The solid blue line represented ROC curve, and the dashed blue line was its 95% confidence interval. The area under the blue solid line (AUC) indicted the predictive power of miR-671-5p (low predictive power: 0.50 < AUC ≤ 0.70, medium predictive power: 0.70< AUC ≤0.90, and high predictive power: 0.9< AUC ≤1.0). ROC, receiver operating characteristic; AUC, the area under the ROC curve; M, distant metastasis; pN, pathologic lymph node metastasis; BCR, biochemical recurrence.

**Figure S3.** Generation of stable cell lines with miR-671 overexpression or knockdown.

We established two PCa cell lines with stable miR-671 overexpression (A) or knockdown (B) by lentivirus infection. The data were presented as Means±SD from three biological replicates. ^***^*P* < 0.001; ^****^*P* < 0.0001; Student’s *t*-test.

**Figure S4.** MiR-671 facilitated C4-2 cells proliferation, migration and invasion in vitro.

Colony formation assays (A), wound healing assays (B), and Transwell assays (C) were performed to assess the proliferation, migration and invasion ability in miR-671 knockdown or overexpressing C4-2 cells. The data were presented as Means±SD from three biological replicates. ^*^*P* < 0.05; ^**^*P* < 0.01; ^***^*P* < 0.001; ^****^*P* < 0.0001; Student’s *t*-test.

**Figure S5.** Effect of miR-671 on apoptosis of PCa cells.

A-C, overexpression or knockdown of miR-671 was not associated with apoptosis in both PC-3 and C4-2 cell lines, as determined by Annexin/PI staining. The data were presented as Means±SD from three biological replicates. ^ns^*P* > 0.05; Student’s *t*-test.

**Figure S6.** The correlation of NFIA or RBMS3 mRNAs expression with miR-671 expression in PCa cells.

A-B, q-PCR was performed to detect NFIA and RBMS3 mRNA expression in PCa cells with stable overexpression or knockdown of miR-671. The data were presented as Means±SD from three biological replicates. ^ns^*P* > 0.05; ^*^*P* < 0.05; Student’s *t*-test.

**Figure S7.** MiR-671 promoted C4-2 cells proliferation, migration and invasion via down-regulating of NFIA.

(A-B) Promotive effect on proliferation, migration, and invasion in C4-2 cells after Knockdown of NFIA expression. (C-D) The proliferative, migratory, and invasive abilities of C4-2 cells impaired after up-regulating NFIA, and the carcinogenesis effect of miR-671 could also be reversed. The data were presented as Means±SD from three biological replicates. ^**^*P* < 0.01; ^***^*P* < 0.001; ^****^*P* < 0.0001; Student’s *t*-test.

**Figure S8.** miR-671 negatively regulated the expression of CRYAB.

(A) The correlations between miR-671 expression and CRYAB expression in GSE21034 and TCGA datasets. (B) Q-PCR analysis was performed to detect the expression changes of CRYAB mRNA after infecting PCa cells with LV-miR-671. (C) CRYAB IHC staining in xenografts with LV-in-miR-671 cells or LV-in-NC cells. Magnification, 200x. Scale bars, 100 μm. The data were presented as Means±SD from three biological replicates. ^*^*P* < 0.05; ^***^*P* < 0.001; ^****^*P* < 0.0001; Student’s *t*-test.

**Figure S9.** MiR-671 promoted tumor development and metastasis via down-regulating of CRYAB.

(A) CRYAB protein expression in rescue experiment of CRYAB overexpression in cells with stable miR-671 overexpression and corresponding control cells. (B-C) The proliferative, migratory, and invasive abilities of PC-3 and C4-2 cells impaired after up-regulating CRYAB, and the carcinogenesis effect of miR-671 could also be reversed. The data were presented as Means±SD from three biological replicates. ^*^*P* < 0.05; ^**^*P* < 0.01; ^***^*P* < 0.001; ^****^*P* < 0.0001; Student’s *t*-test.

**Figure S10.** NFIA was down-regulated in metastatic PCa tissues, and low expression of NFIA indicated poor prognosis in GSE21034.

(A) The expression levels of NFIA decreased steadily from adjacent normal tissues, primary localized PCa tissues, to metastatic PCa tissues in GSE21034. (C-F) NFIA expression levels in PCa tissues with different tumor stage, lymph node metastasis status, Gleason score and BCR status. (G-H) Kaplan–Meier analysis of overall survival curves and BCR-free survival curves of PCa patients with different NFIA expression levels. The median of NFIA expression was used to stratify the samples. ^ns^*P* > 0.05; ^*^*P* < 0.05; ^**^*P* < 0.01; ^***^*P* < 0.001; ^****^*P* < 0.0001; Student’s *t*-test. cT, clinical tumor stage; pT, pathologic tumor stage; pN, pathologic lymph node metastasis; GS, Gleason score; BCR, biochemical recurrence; HR, hazard ratio.

**Figure S11.** CRYAB was down-regulated in metastatic PCa tissues, and low expression of CRYAB indicated poor prognosis in GSE21034.

(A) The expression levels of CRYAB decreased steadily from adjacent normal tissues, primary localized PCa tissues, to metastatic PCa tissues in GSE21034. (C-F) CRYAB expression levels in PCa tissues with different tumor stage, lymph node metastasis status, Gleason score and BCR status. (G-H) Kaplan–Meier analysis of overall survival curves and BCR-free survival curves of PCa patients with different CRYAB expression levels. The median of CRYAB expression was used to stratify the samples. ^ns^*P* > 0.05; ^*^*P* < 0.05; ^**^*P* < 0.01; ^***^*P* < 0.001; ^****^*P* < 0.0001; Student’s *t*-test. cT, clinical tumor stage; pT, pathologic tumor stage; pN, pathologic lymph node metastasis; GS, Gleason score; BCR, biochemical recurrence; HR, hazard ratio.

**Figure S12.** High expression of NFIA or CRYAB negatively correlated with advanced clinicopathological characteristics, and their low expression indicated poor prognosis in TCGA.

(A-D and G-J) NFIA or CRYAB expression levels in PCa tissues with different tumor stage, lymph node metastasis status, Gleason score and BCR status. (E-F and K-L) Kaplan–Meier analysis of overall survival curves and BCR-free survival curves of PCa patients with different NFIA or CRYAB expression levels. The median of NFIA or CRYAB expression was used to stratify the samples. ^ns^*P* > 0.05; ^*^*P* < 0.05; ^**^*P* < 0.01; ^***^*P* < 0.001; ^****^*P* < 0.0001; Student’s *t*-test. pT, pathologic tumor stage; pN, pathologic lymph node metastasis; GS, Gleason score; BCR, biochemical recurrence; HR, hazard ratio.

**Figure S13.** Validation of NFIA protein from the HPA database.

NFIA protein expression was moderate in the 3/3 of the adjacent normal tissues (A-B), moderate in the 3/12 PCa tissues (C-D), low in the 7/12 PCa tissues (E-F), and negative in the 2/12 PCa tissues (G-H).

**Supplementary Table Legends**

**Table S1.** MiRNAs related to PCa metastasis identified by bioinformatics analysis.

ANT, adjacent normal tissues; PPCa, primary localized PCa tissues; MPCa, metastatic PCa tissues; FC, fold change.

**Table S2.** Prognostic value of miRNAs in BCR-free survival

BCR, biochemical recurrence; HR, hazard ratio; CI, confidence intervals.

**Table S3.** Identifying the expression of miR-671 in other tumors by dbDEMC 2.0

**Table S4.** Univariate and multivariate Cox regression analysis for biochemical recurrence-free survival in TCGA

Age, between age≤62 and age>62; pT, pathologic tumor stage between T2 and T3-4; pN, pathologic regional lymph node metastasis, between N0 and N1; Gleason score, among Gleason score≤7 and >7; miR-671-5p, continuous miR-671-5p expression levels; HR, Hazard ratio; CI, confidence interval.

**Table S5.** miR-671-5p was a potential diagnostic and prognostic marker

AUC, area under the curve; CI, confidence interval; BCR, biochemical recurrence; pN, pathologic regional lymph node metastasis; M, distant metastasis.

**Table S6.** The correlations between NFIA expression and its target genes expression in PCa (cBioPortal).

ANT, adjacent normal tissues.

**Table S7.** Univariate and multivariate Cox regression analysis for biochemical recurrence-free survival in GSE21034 (NFIA)

Age, between age≤62 and age>62; pT, pathologic tumor stage between T2 and T3-4; pN, pathologic regional lymph node metastasis, between N0 and N1; Gleason score, among Gleason score≤7 and >7; NFIA, continuous NFIA expression levels. HR, Hazard ratio; CI, confidence interval.

**Table S8.** Univariate and multivariate Cox regression analysis for biochemical recurrence-free survival in GSE21034 (CRYAB)

Age, between age≤62 and age>62; pT, pathologic tumor stage between T2 and T3-4; pN, pathologic regional lymph node metastasis, between N0 and N1; Gleason score, among Gleason score≤7 and >7; CRYAB, continuous CRYAB expression levels. HR, Hazard ratio; CI, confidence interval.

**Table S9.** Univariate and multivariate Cox regression analysis for biochemical recurrence-free survival in TCGA (NFIA)

Age, between age≤62 and age>62; pT, pathologic tumor stage between T2 and T3-4; pN, pathologic regional lymph node metastasis, between N0 and N1; Gleason score, among Gleason score≤7 and >7; NFIA, continuous NFIA expression levels. HR, Hazard ratio; CI, confidence interval.

**Table S10.** Univariate and multivariate Cox regression analysis for biochemical recurrence-free survival in TCGA (CRYAB)

Age, between age≤62 and age>62; pT, pathologic tumor stage between T2 and T3-4; pN, pathologic regional lymph node metastasis, between N0 and N1; Gleason score, among Gleason score≤7 and >7; CRYAB, continuous CRYAB expression levels. HR, Hazard ratio; CI, confidence interval.

**Table S11.** Basic information of included datasets

ID, identification; n, number; ANT, adjacent normal tissues; P, primary localized PCa tissues; M, metastatic PCa tissues.

**Table S12.** Primer information used in the study
