# Supplementary figures and images for: MiRNA-671-5p Promotes prostate cancer development and metastasis by targeting NFIA/CRYAB axis
Source: Cell Death Dis. 2020 Nov 3;11(11):949. doi: 10.1038/s41419-020-03138-w (PMC7642259; doi:10.1038/s41419-020-03138-w)

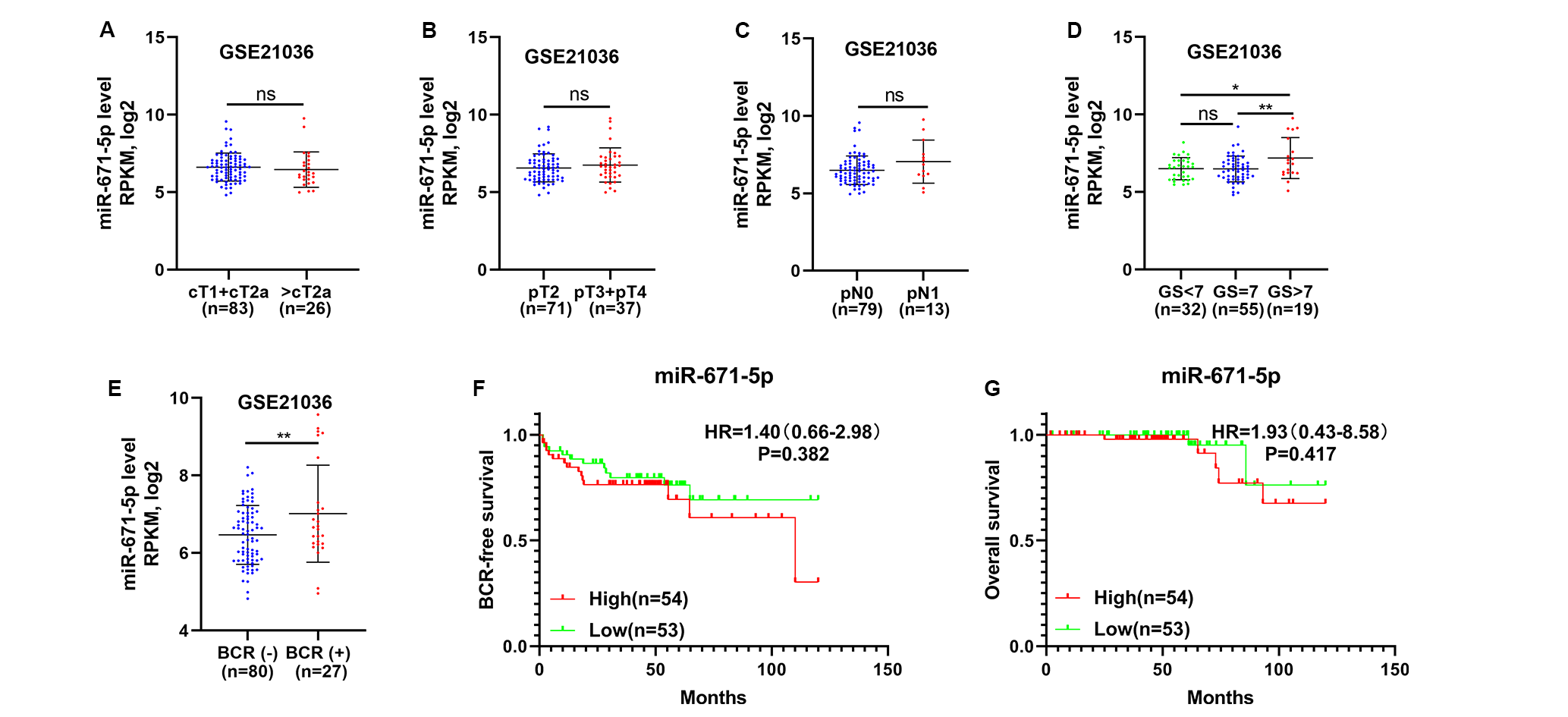

Supplement: Supplementary file 3 — Figure S1 [file 41419_2020_3138_MOESM3_ESM.tif]

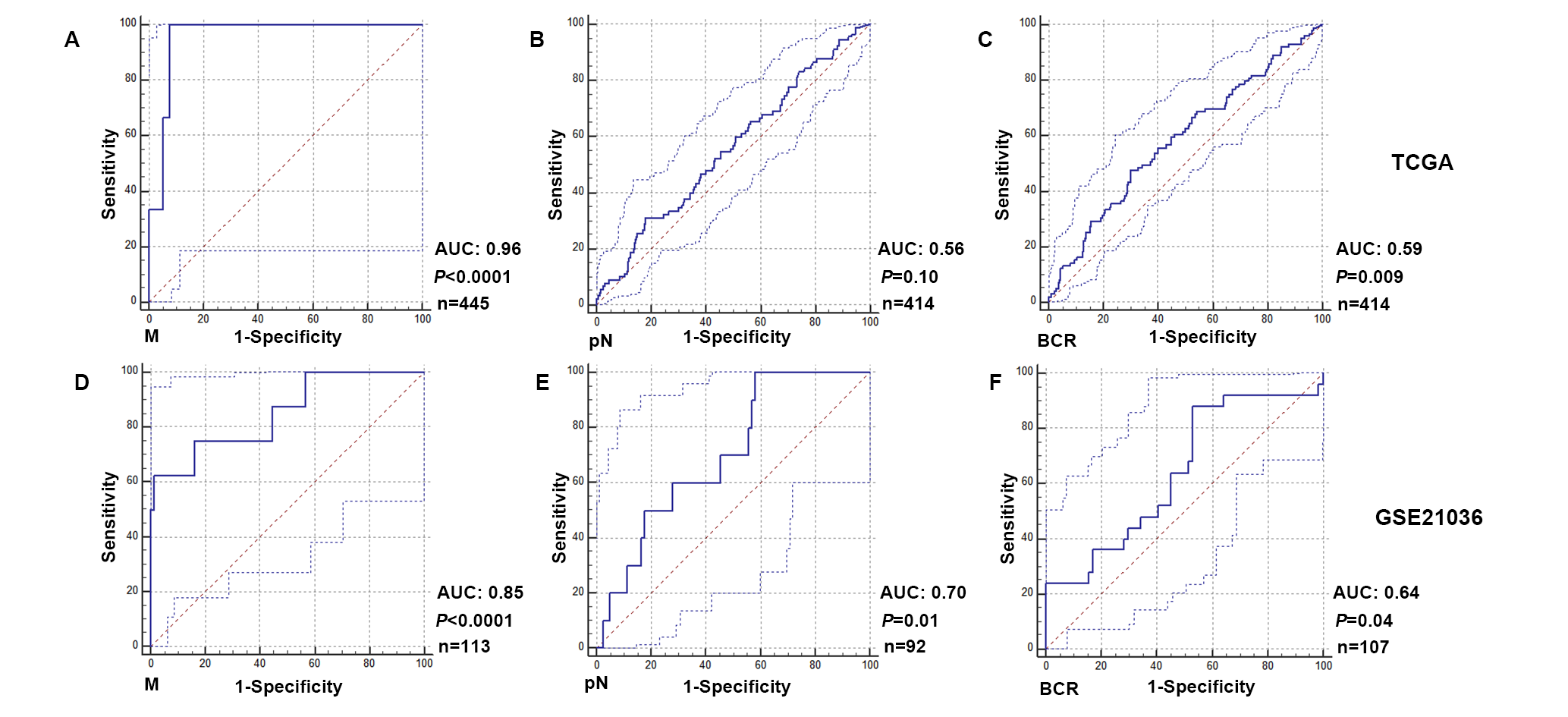

Supplement: Supplementary file 4 — Figure S2 [file 41419_2020_3138_MOESM4_ESM.tif]

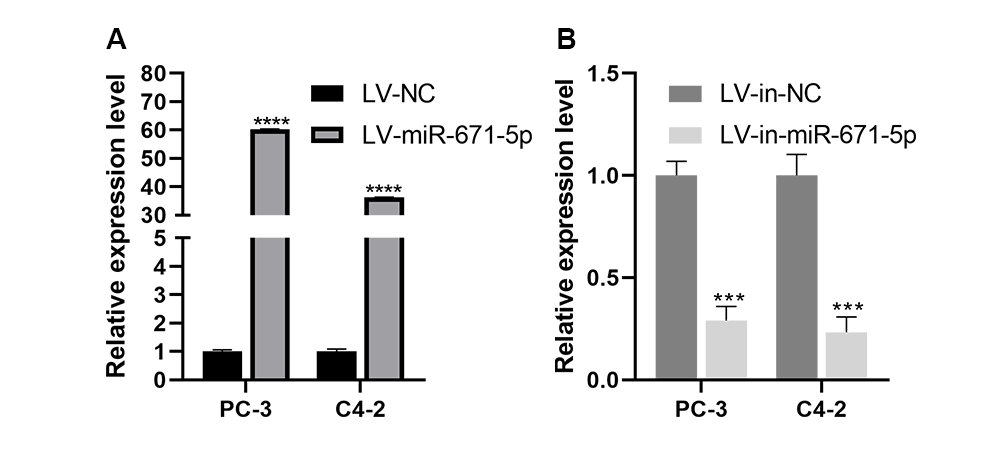

Supplement: Supplementary file 5 — Figure S3 [file 41419_2020_3138_MOESM5_ESM.tif]

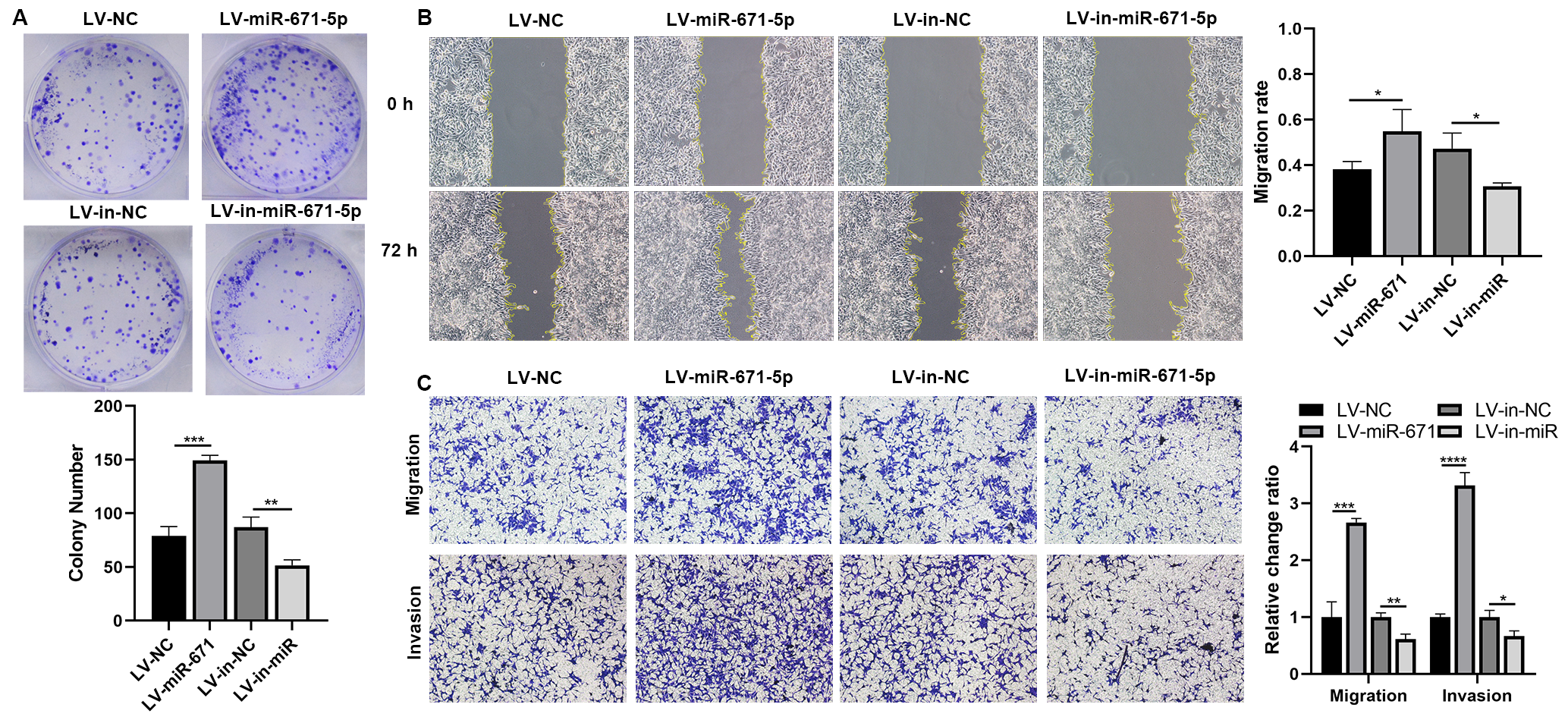

Supplement: Supplementary file 6 — Figure S4 [file 41419_2020_3138_MOESM6_ESM.tif]

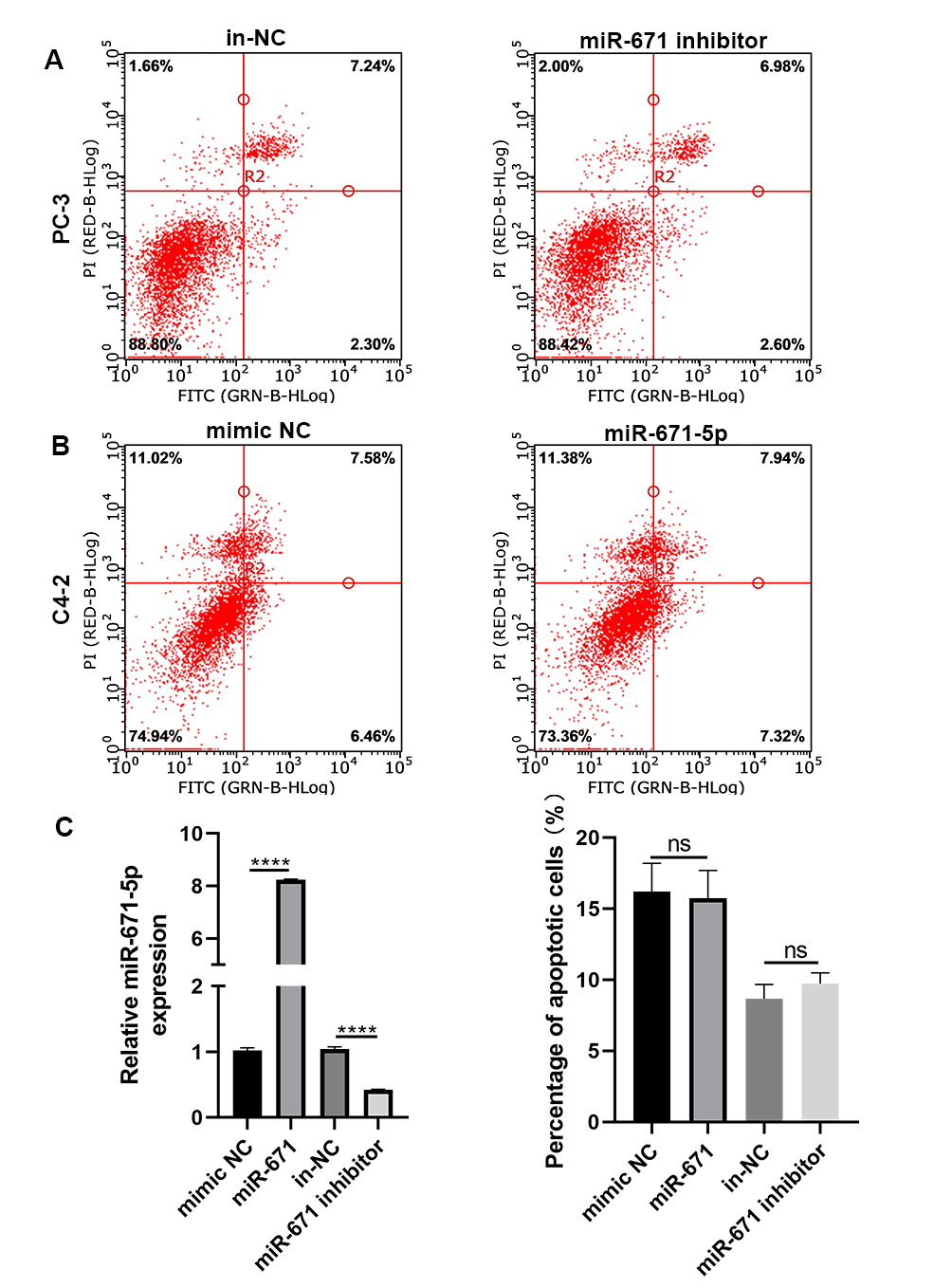

Supplement: Supplementary file 7 — Figure S5 [file 41419_2020_3138_MOESM7_ESM.tif]

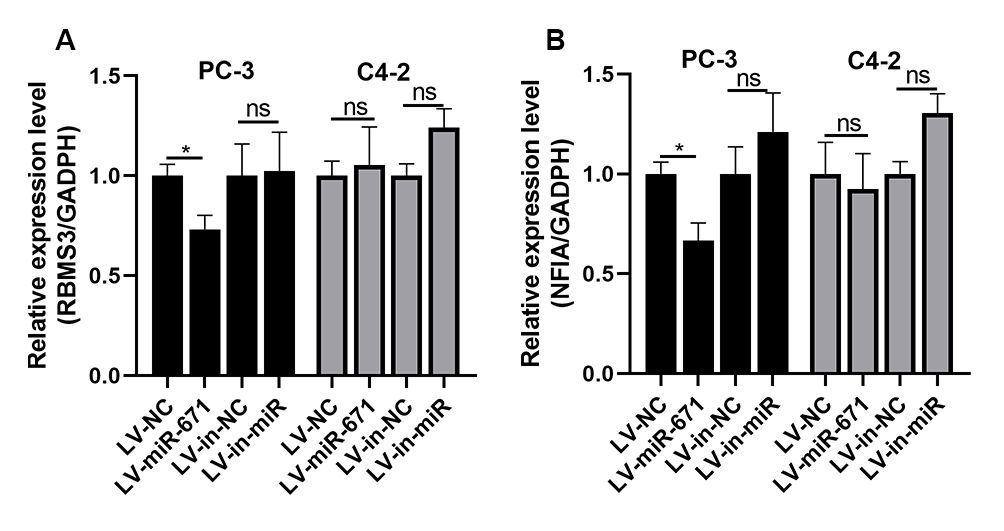

Supplement: Supplementary file 8 — Figure S6 [file 41419_2020_3138_MOESM8_ESM.tif]

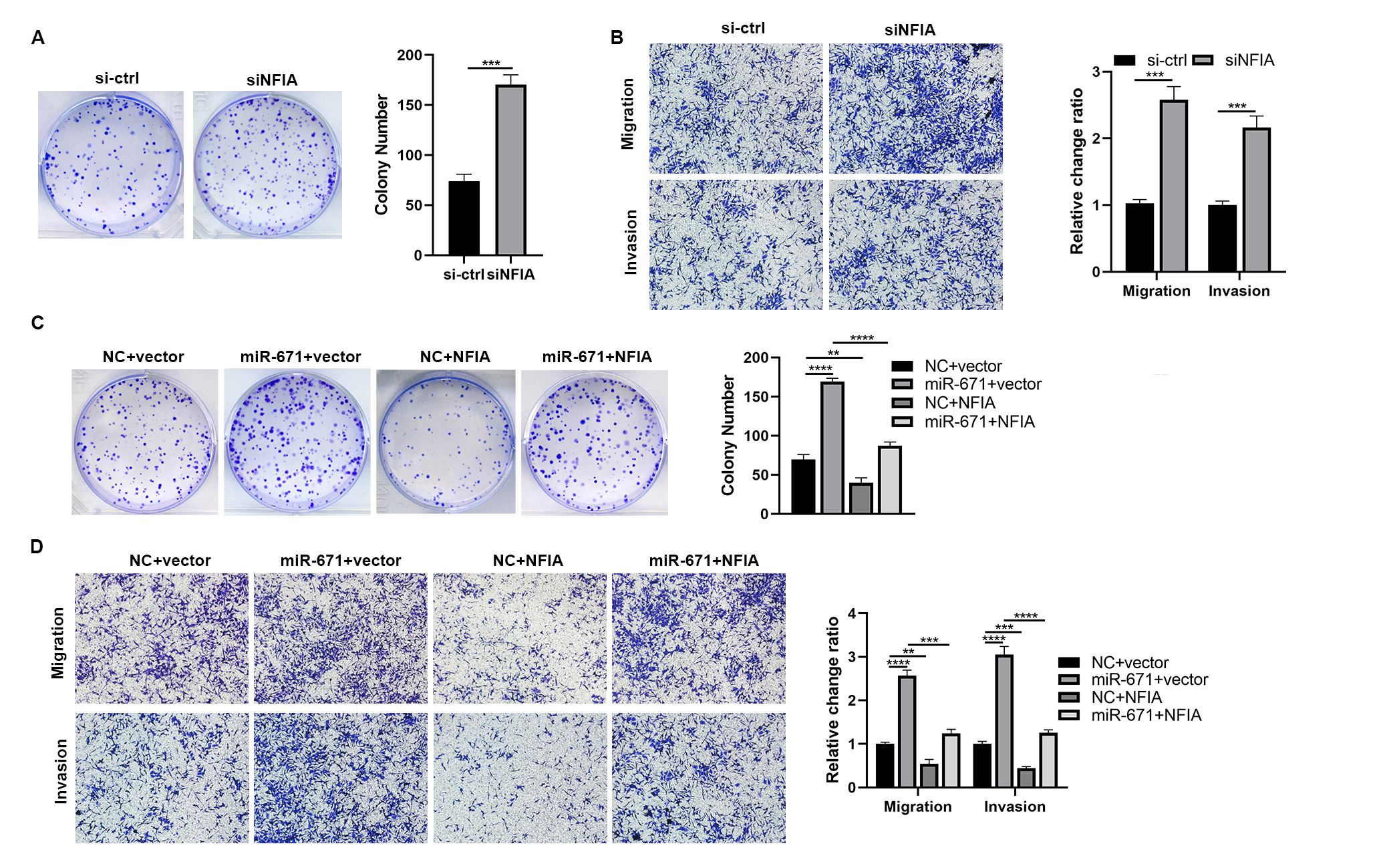

Supplement: Supplementary file 9 — Figure S7 [file 41419_2020_3138_MOESM9_ESM.tif]

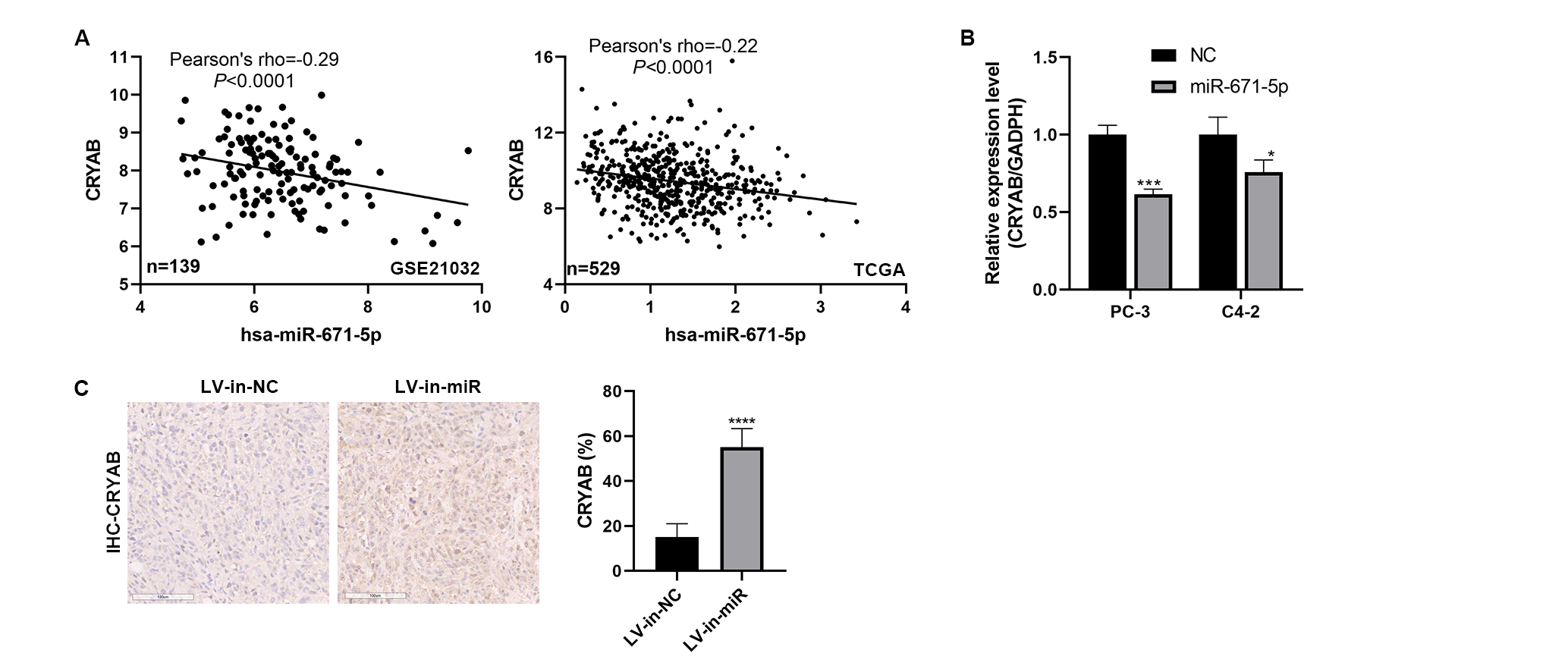

Supplement: Supplementary file 10 — Figure S8 [file 41419_2020_3138_MOESM10_ESM.tif]

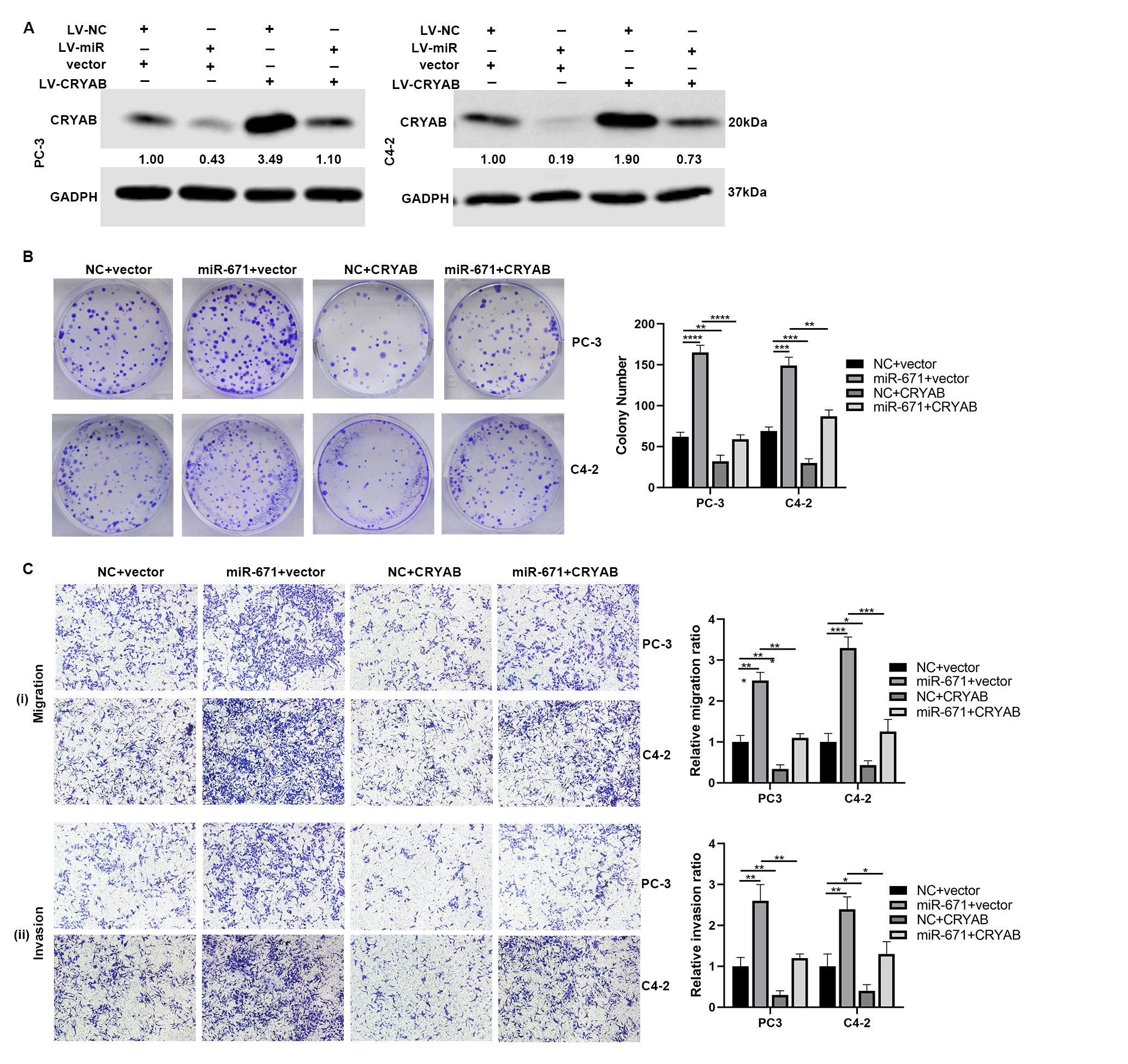

Supplement: Supplementary file 11 — Figure S9 [file 41419_2020_3138_MOESM11_ESM.tif]

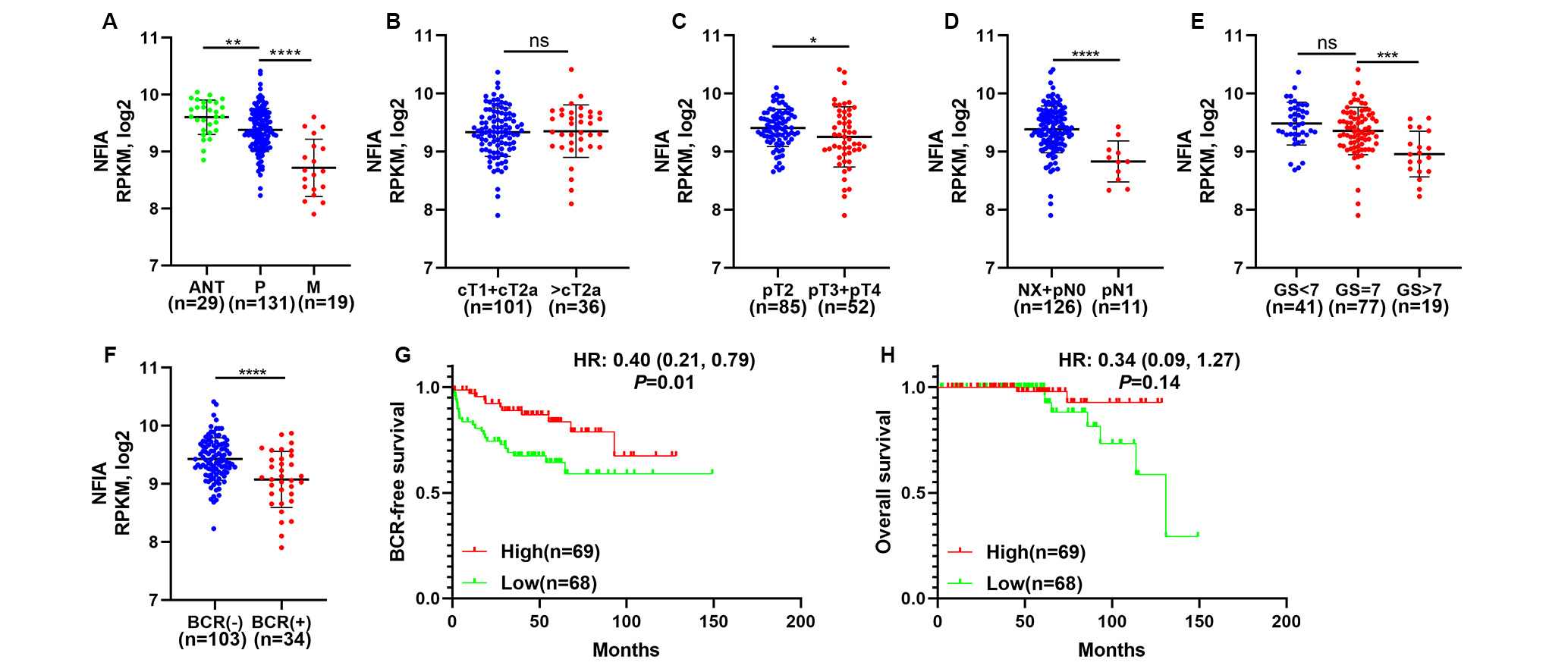

Supplement: Supplementary file 12 — Figure S10 [file 41419_2020_3138_MOESM12_ESM.tif]

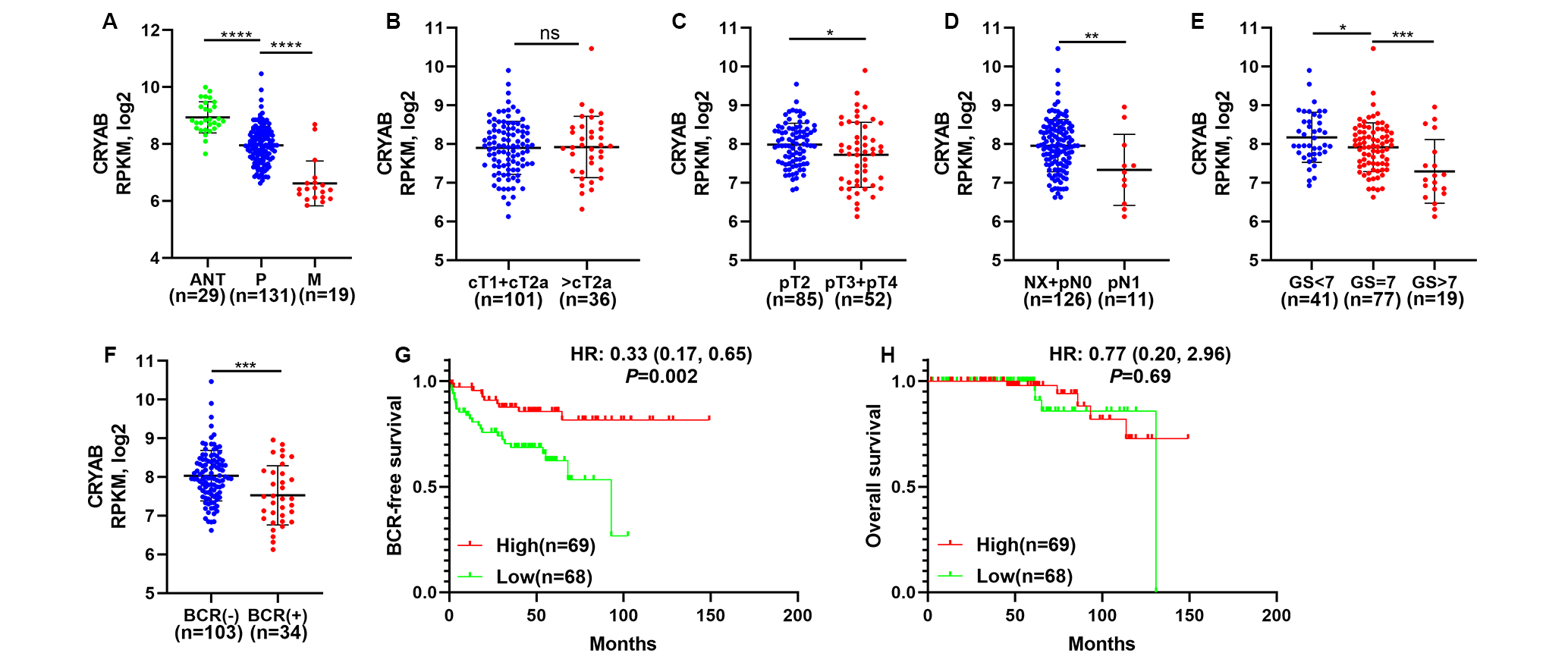

Supplement: Supplementary file 13 — Figure S11 [file 41419_2020_3138_MOESM13_ESM.tif]

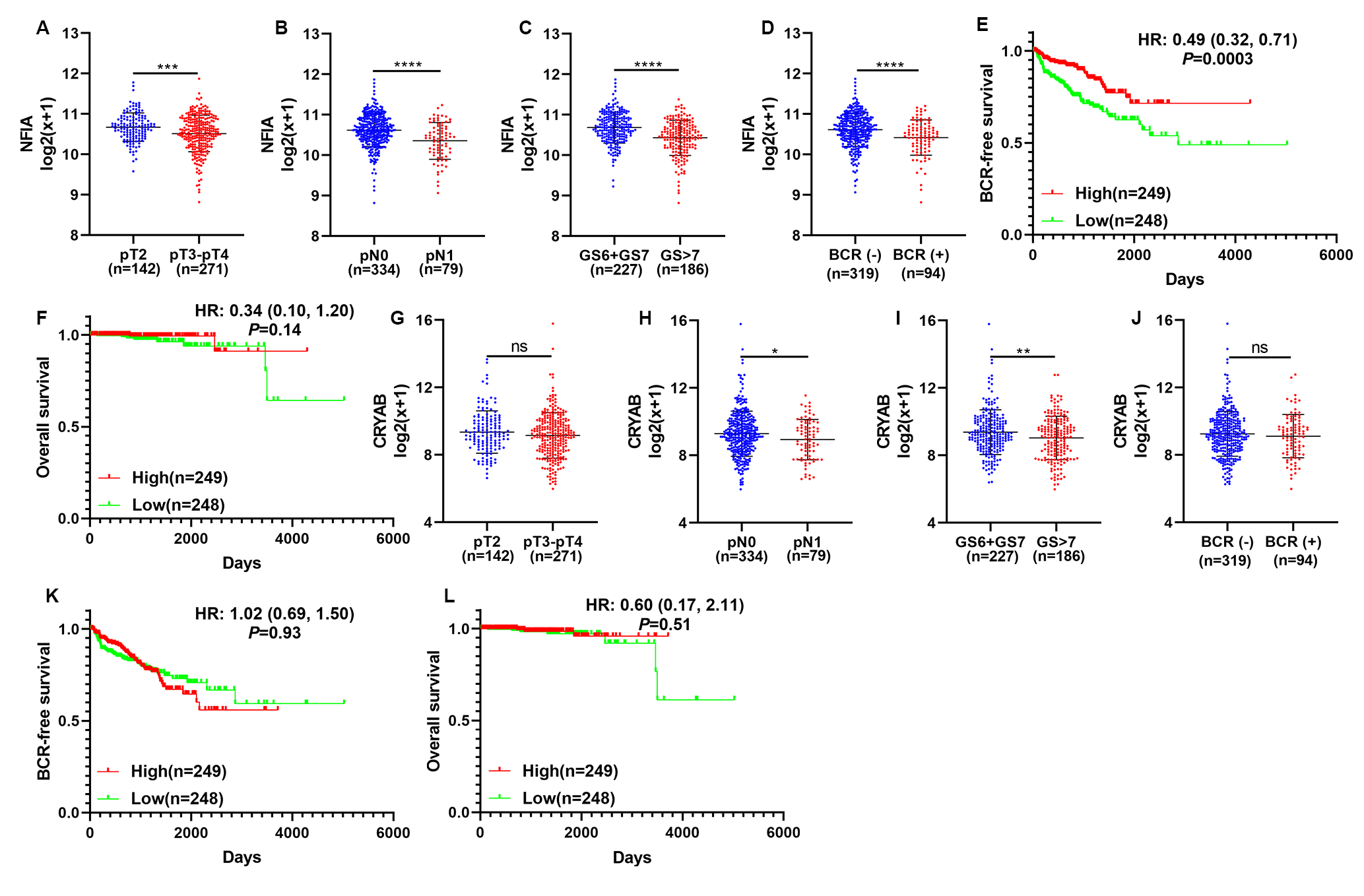

Supplement: Supplementary file 14 — Figure S12 [file 41419_2020_3138_MOESM14_ESM.tif]

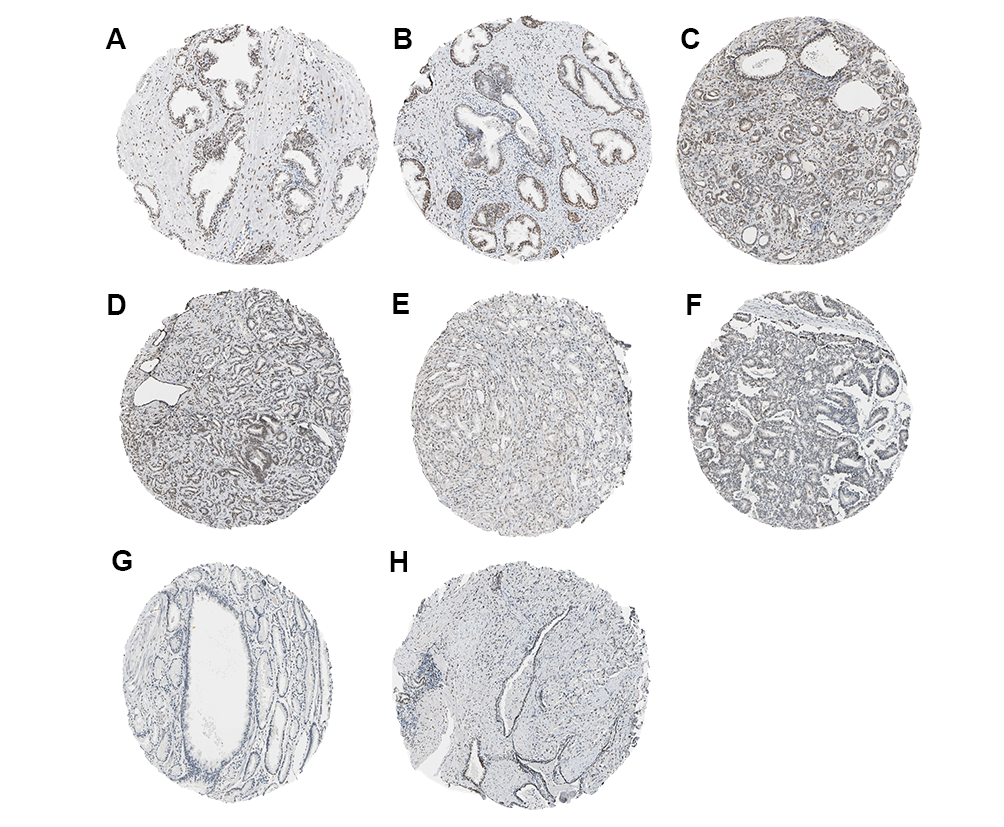

Supplement: Supplementary file 15 — Figure S13 [file 41419_2020_3138_MOESM15_ESM.tif]
