# Supplementary material for: MiRNA-671-5p Promotes prostate cancer development and metastasis by targeting NFIA/CRYAB axis
Source: Cell Death Dis. 2020 Nov 3;11(11):949. doi: 10.1038/s41419-020-03138-w (PMC7642259; doi:10.1038/s41419-020-03138-w)
Supplement: Supplementary file 16 — Table S1 [file 41419_2020_3138_MOESM16_ESM.docx]

**Table S1.** MiRNAs related to PCa metastasis identified by bioinformatics analysis.

| miRNAs | Tumor vs. ANT | | MPCa vs. PPCa | |
| --- | --- | --- | --- | --- |
|  | FC | adj.*P*.Val | FC | adj.*P*.Val |
| hsa-miR-877-5p | 1.84377655 | 0.000213221 | 1.8427638 | 0.004251099 |
| hsa-miR-671-5p | 1.57598485 | 0.004214354 | 2.265501 | 0.0000741 |
| hsa-miR-629-5p | 1.60601305 | 9.27E-07 | 1.6752502 | 1.40E-05 |
| hsa-miR-627 | 1.67600561 | 0.000039 | 1.8754524 | 0.0000788 |
| hsa-miR-550a-5p | 1.72218885 | 0.000188423 | 2.679662 | 6.87E-08 |
| hsa-miR-501-3p | 1.53907857 | 0.00033387 | 1.5166006 | 0.009969568 |
| hsa-miR-345-5p | 1.60638156 | 0.0069279 | 2.2941511 | 0.000382781 |
| hsa-miR-130b-3p | 1.62902691 | 1.17E-05 | 1.7275779 | 0.000130404 |
| hsa-miR-663 | 2.66977467 | 2.51E-09 | 2.046377 | 0.000361468 |
| hsa-miR-10b-3p | 1.68526556 | 0.002280304 | 1.7459958 | 0.012829263 |
| hsa-miR-622 | 1.67802498 | 0.001480194 | 1.8986144 | 0.002446471 |
| hsa-miR-518c-5p | 1.84252328 | 0.000664024 | 1.7318996 | 0.022241312 |
| hsa-miR-1225-5p | 1.74317161 | 0.000220396 | 1.8742521 | 0.000814272 |
| hsa-miR-575 | 1.78187968 | 0.000145948 | 2.5058263 | 0.00000337 |
| hsa-miR-486-5p | 1.65680397 | 0.003939217 | 4.6397783 | 1.03E-13 |
| hsa-miR-33b | 1.72268833 | 0.002354302 | 3.2312871 | 5.34E-08 |
| hsa-miR-135a-3p | 2.21965582 | 1.17E-05 | 2.1565167 | 0.001225962 |
| hsa-miR-548c-3p | 2.39533865 | 0.000017 | 3.2433507 | 0.00000337 |
| hsa-miR-526b | 1.50116028 | 0.022664547 | 1.9972421 | 0.002791578 |
| hsa-miR-648 | 1.91506915 | 0.0000648 | 1.7286479 | 0.010778885 |
| hsa-miR-150-3p | 1.74027948 | 6.48E-05 | 1.9229266 | 0.000178072 |
| hsa-miR-602 | 1.78017837 | 0.00000256 | 2.1934537 | 8.65E-08 |
| hsa-miR-451 | 1.619504 | 0.017642727 | 2.4436073 | 0.000890775 |
| hsa-miR-512-3p | 1.49768548 | 0.002669159 | 1.7804068 | 0.000907524 |
| hsa-miR-198 | 1.55358969 | 0.009281329 | 2.3889481 | 0.0000757 |
| hsa-miR-659 | 1.97433648 | 0.000017 | 1.8843411 | 0.001989674 |
| hsa-miR-936 | 1.72238339 | 0.000278121 | 1.8633798 | 0.001766689 |
| hsa-miR-455-5p | 0.55603176 | 0.0000258 | 0.5119406 | 0.000372799 |
| hsa-miR-27b-3p | 0.53944748 | 5.28E-06 | 0.2961073 | 1.20E-14 |
| hsa-miR-24-3p | 0.65572483 | 5.74E-06 | 0.4998735 | 1.10E-09 |
| hsa-miR-23b-3p | 0.54966878 | 2.76E-05 | 0.2596752 | 2.72E-16 |
| hsa-miR-222-3p | 0.30787988 | 9.43E-10 | 0.272811 | 1.36E-08 |
| hsa-miR-221-5p | 0.54647743 | 0.000410097 | 0.1885912 | 6.54E-16 |
| hsa-miR-205-5p | 0.13541184 | 0.00022429 | 0.01071 | 2.56E-11 |
| hsa-miR-204-5p | 0.4042962 | 1.73E-05 | 0.2349418 | 3.39E-08 |
| hsa-miR-152 | 0.59544239 | 0.000000332 | 0.4949566 | 3.62E-08 |
| hsa-miR-145-3p | 0.43495678 | 4.10E-05 | 0.0990883 | 3.98E-27 |
| hsa-miR-143-5p | 0.34829482 | 1.46E-05 | 0.0607102 | 9.29E-29 |
| hsa-miR-1-3p | 0.3978925 | 0.004114553 | 0.0229409 | 1.09E-30 |
| hsa-miR-133b | 0.34236506 | 0.000041 | 0.0481345 | 9.29E-29 |
| hsa-miR-133a | 0.33682986 | 6.94E-08 | 0.1239615 | 1.94E-21 |
| hsa-miR-125b | 0.664176 | 0.003044892 | 0.2733353 | 8.85E-16 |
| hsa-miR-31-5p | 0.34519849 | 7.01E-07 | 0.3750254 | 0.000639916 |
| hsa-miR-455-3p | 0.64213325 | 0.00804541 | 0.4221374 | 0.000101054 |
| hsa-miR-130a | 0.61936514 | 0.000659378 | 0.2561597 | 2.39E-17 |
| hsa-miR-338-3p | 0.55026134 | 0.000174229 | 0.550666 | 0.004965456 |
| hsa-miR-24-1-5p | 0.55057096 | 1.15E-05 | 0.2992811 | 5.16E-15 |
| hsa-miR-224 | 0.39111498 | 7.81E-08 | 0.5794846 | 0.01397806 |
| hsa-miR-139-5p | 0.56167661 | 2.72E-08 | 0.6362241 | 0.00048768 |
| hsa-miR-100 | 0.60342886 | 0.00124587 | 0.2293845 | 6.54E-16 |
| hsa-miR-886-3p | 0.44292152 | 0.000000446 | 0.3985489 | 0.00000589 |

ANT, adjacent normal tissues; PPCa, primary localized PCa tissues; MPCa, metastatic PCa tissues; FC, fold change.
