# Supplementary material for: MiRNA-671-5p Promotes prostate cancer development and metastasis by targeting NFIA/CRYAB axis
Source: Cell Death Dis. 2020 Nov 3;11(11):949. doi: 10.1038/s41419-020-03138-w (PMC7642259; doi:10.1038/s41419-020-03138-w)
Supplement: Supplementary file 17 — Table S2 [file 41419_2020_3138_MOESM17_ESM.docx]

Table S2. Prognostic value of miRNAs in BCR-free survival

| miRNAs | GSE21036 | | | TCGA | | |
| --- | --- | --- | --- | --- | --- | --- |
|  | HR | 95% CI | *P* | HR | 95% CI | *P* |
| miR-671-5p | 1.40 | 0.66, 2.98 | 0.38 | 1.83 | 1.23, 2.72 | 0.003 |
| miR-130b-3p | 6.18 | 2.88, 13.29 | <0.0001 | 1.30 | 0.88, 1.93 | 0.19 |
| miR-221-5p | 0.20 | 0.09, 0.44 | 0.0001 | 0.46 | 0.31, 0.68 | 0.0002 |
| miR-133b | 0.47 | 0.22, 0.99 | 0.05 | 0.45 | 0.30, 0.67 | 0.0001 |
| miR-455-5p | 0.33 | 0.15, 0.70 | 0.01 | 0.80 | 0.54, 1.19 | 0.28 |
| miR-27b-3p | 0.32 | 0.15, 0.69 | 0.004 | 1.33 | 0.99, 1.98 | 0.16 |
| miR-145-3p | 0.25 | 0.11, 0.53 | 0.0004 | 0.69 | 0.46, 1.02 | 0.06 |
| miR-23b-3p | 0.41 | 0.19, 0.87 | 0.02 | 1.00 | 0.068, 1.49 | 0.99 |
| miR-1-3p | 0.16 | 0.07, 0.34 | <0.0001 | 1.41 | 0.66, 3.01 | 0.18 |
| miR-204-5P | 0.25 | 0.11, 0.53 | 0.0004 | 0.71 | 0.48, 1.05 | 0.09 |
| miR-205-5p | 0.37 | 0.18, 0.80 | 0.02 | 0.93 | 0.63, 1.38 | 0.72 |
| miR-133a-3p | 0.49 | 0.23, 1.05 | 0.07 | 0.83 | 0.56, 1.23 | 0.35 |
| miR-222-3p | 0.82 | 0.39, 1.76 | 0.62 | 0.61 | 0.41, 0.90 | 0.01 |

BCR, biochemical recurrence; HR, hazard ratio; CI, confidence intervals.
