# Supplementary material for: MiRNA-671-5p Promotes prostate cancer development and metastasis by targeting NFIA/CRYAB axis
Source: Cell Death Dis. 2020 Nov 3;11(11):949. doi: 10.1038/s41419-020-03138-w (PMC7642259; doi:10.1038/s41419-020-03138-w)
Supplement: Supplementary file 18 — Table S3 [file 41419_2020_3138_MOESM18_ESM.docx]

Table S3. Identifying the expression of miR-671 in other tumors by dbDEMC 2.0

| **ID** | **Cancer Type** | **Cancer Subtype** | **Design** | **logFC** | **Status** |
| --- | --- | --- | --- | --- | --- |
| TCGA_BRCA | breast cancer | breast invasive carcinoma | cancer vs normal | 0.48 | UP |
| TCGA_BRCA | breast cancer | breast invasive carcinoma | high grade vs low grade | 0.1 | UP |
| TCGA_KIRP | kidney cancer | kidney renal papillary cell carcinoma | cancer vs normal | 0.99 | UP |
| TCGA_KIRC | kidney cancer | kidney renal clear cell carcinoma | cancer vs normal | 0.33 | UP |
| TCGA_KIRC | kidney cancer | kidney renal clear cell carcinoma | high grade (Ⅲ) vs low grade (Ⅰ) | 0.2 | UP |
| TCGA_KIRC | kidney cancer | kidney renal clear cell carcinoma | high grade (Ⅳ) vs low grade (Ⅰ) | 0.34 | UP |
| TCGA_KICH | kidney cancer | kidney chromophobe cancer | cancer vs normal | 0.59 | UP |
| TCGA_LUAD | lung cancer | lung adenocarcinoma | cancer vs normal | 0.67 | UP |
| TCGA_LUSC | lung cancer | lung squamous cell carcinoma | cancer vs normal | 0.77 | UP |
| TCGA_UCEC | endometrial cancer | uterine corpus endometrial carcinoma | cancer vs normal | 0.78 | UP |
| TCGA_COAD | colon cancer | colon adenocarcinoma | cancer vs normal | 0.56 | UP |
| TCGA_HNSC | head and neck cancer | head and neck squamous cell carcinoma | cancer vs normal | 0.88 | UP |
| TCGA_THCA | thyroid cancer | thyroid carcinoma | cancer vs normal | 0.49 | UP |
| TCGA_PRAD | prostate cancer | prostate adenocarcinoma | cancer vs normal | 0.25 | UP |
| TCGA_STAD | gastric cancer | stomach adenocarcinoma | cancer vs normal | 0.49 | UP |
| TCGA_LIHC | hepatocellular carcinoma | hepatocellular carcinoma | cancer vs normal | 0.61 | UP |
| TCGA_BLCA | bladder cancer | bladder urothelial carcinoma | cancer vs normal | 1 | UP |
| TCGA_SKCM | melanoma | skin cutaneous melanoma | metastasis | -0.28 | DOWN |
| TCGA_BLCA | bladder cancer | bladder urothelial carcinoma | high grade vs low grade | -0.27 | DOWN |
