# Supplementary material for: MiRNA-671-5p Promotes prostate cancer development and metastasis by targeting NFIA/CRYAB axis
Source: Cell Death Dis. 2020 Nov 3;11(11):949. doi: 10.1038/s41419-020-03138-w (PMC7642259; doi:10.1038/s41419-020-03138-w)
Supplement: Supplementary file 19 — Table S4 [file 41419_2020_3138_MOESM19_ESM.doc]

**Table S4.** Univariate and multivariate Cox regression analysis for biochemical recurrence-free survival in TCGA

|  | Univariate Cox regression analysis | |  | Multivariate Cox regression analysis | |
| --- | --- | --- | --- | --- | --- |
|  | HR (95% CI) | *P* |  | HR (95% CI) | *P* |
| Age | 1.15 (0.77, 1.73) | 0.49 |  | 0.99 (0.66, 1.50) | 0.97 |
| pT | 4.66 (2.48, 8.76) | <0.0001 |  | 3.00 (1.54, 5.86) | 0.001 |
| pN | 2.21 (1.42, 3.45) | <0.0001 |  | 1.07 (0.67, 1.70) | 0.79 |
| Gleason score | 4.34 (2.73, 6.90) | <0.0001 |  | 2.95 (1.78, 4.90) | <0.0001 |
| miR-671-5p | 1.73 (1.14, 2.61) | 0.01 |  | 1.61 (1.05, 2.45) | 0.03 |

Age, between age≤62 and age>62; pT, pathologic tumor stage between T2 and T3-4; pN, pathologic regional lymph node metastasis, between N0 and N1; Gleason score, among Gleason score≤7 and >7; miR-671-5p, continuous miR-671-5p expression levels; HR, Hazard ratio; CI, confidence interval.
