# Supplementary material for: MiRNA-671-5p Promotes prostate cancer development and metastasis by targeting NFIA/CRYAB axis
Source: Cell Death Dis. 2020 Nov 3;11(11):949. doi: 10.1038/s41419-020-03138-w (PMC7642259; doi:10.1038/s41419-020-03138-w)
Supplement: Supplementary file 20 — Table S5 [file 41419_2020_3138_MOESM20_ESM.doc]

**Table S5.** miR-671-5p was a potential diagnostic and prognostic marker

|  | TCGA | |  | GSE21036 | |
| --- | --- | --- | --- | --- | --- |
|  | AUC (95% CI) | *P* |  | AUC (95% CI) | *P* |
| BCR | 0.59 (0.54, 0.63) | 0.009 |  | 0.64 (0.53, 0.74) | 0.04 |
| pN | 0.56 (0.51, 0.60) | 0.10 |  | 0.70 (0.60, 0.80) | 0.01 |
| M | 0.96 (0.93, 0.97) | <0.0001 |  | 0.85 (0.76, 0.92) | <0.0001 |

AUC, area under the curve; CI, confidence interval; BCR, biochemical recurrence; pN, pathologic regional lymph node metastasis; M, distant metastasis.
