# Supplementary material for: MiRNA-671-5p Promotes prostate cancer development and metastasis by targeting NFIA/CRYAB axis
Source: Cell Death Dis. 2020 Nov 3;11(11):949. doi: 10.1038/s41419-020-03138-w (PMC7642259; doi:10.1038/s41419-020-03138-w)
Supplement: Supplementary file 21 — Table S6 [file 41419_2020_3138_MOESM21_ESM.docx]

Table S6. The correlations between NFIA expression and its target genes expression in PCa (cBioPortal).

| Genes | Tumor vs. ANT | Pearson’s rho |
| --- | --- | --- |
| MPPED2 | Down-Regulated | 0.42 |
| CNN1 | Down-Regulated | 0.63 |
| CRYAB | Down-Regulated | 0.59 |
| ACTC1 | Down-Regulated | 0.43 |
| KRT15 | Down-Regulated | 0.42 |
| DES | Down-Regulated | 0.61 |
| GABRE | Down-Regulated | 0.43 |
| MAOB | Down-Regulated | 0.53 |
| CLU | Down-Regulated | 0.54 |
| TGM4 | Down-Regulated | 0.02 |
| KRT5 | Down-Regulated | 0.38 |

ANT, adjacent normal tissues.
