# Supplementary material for: MiRNA-671-5p Promotes prostate cancer development and metastasis by targeting NFIA/CRYAB axis
Source: Cell Death Dis. 2020 Nov 3;11(11):949. doi: 10.1038/s41419-020-03138-w (PMC7642259; doi:10.1038/s41419-020-03138-w)
Supplement: Supplementary file 22 — Table S7 [file 41419_2020_3138_MOESM22_ESM.docx]

**Table S7.** Univariate and multivariate Cox regression analysis for biochemical recurrence-free survival in GSE21034 (NFIA)

|  | Univariate Cox regression analysis | |  | Multivariate Cox regression analysis | |
| --- | --- | --- | --- | --- | --- |
|  | HR (95% CI) | *P* |  | HR (95% CI) | *P* |
| Age | 1.60 (0.81, 3.08) | 0.18 |  | 0.92 (0.43, 1.97) | 0.83 |
| pT | 4.92 (2.39, 10.13) | <0.0001 |  | 2.68 (1.20, 6.04) | 0.02 |
| pN | 12.46 (5.84, 26.59) | <0.0001 |  | 4.76 (1.83, 12.38) | 0.001 |
| Gleason score | 7.85 (1.88, 32.76) | 0.005 |  | 4.83 (1.13, 20.72) | 0.03 |
| NFIA | 0.40 (0.20, 0.83) | 0.01 |  | 0.77 (0.35, 1.72) | 0.53 |

Age, between age≤62 and age>62; pT, pathologic tumor stage between T2 and T3-4; pN, pathologic regional lymph node metastasis, between N0 and N1; Gleason score, among Gleason score≤7 and >7; NFIA, continuous NFIA expression levels. HR, Hazard ratio; CI, confidence interval.
