# Supplementary material for: MiRNA-671-5p Promotes prostate cancer development and metastasis by targeting NFIA/CRYAB axis
Source: Cell Death Dis. 2020 Nov 3;11(11):949. doi: 10.1038/s41419-020-03138-w (PMC7642259; doi:10.1038/s41419-020-03138-w)
Supplement: Supplementary file 23 — Table S8 [file 41419_2020_3138_MOESM23_ESM.docx]

**Table S8.** Univariate and multivariate Cox regression analysis for biochemical recurrence-free survival in GSE21034 (CRYAB)

|  | Univariate Cox regression analysis | |  | Multivariate Cox regression analysis | |
| --- | --- | --- | --- | --- | --- |
|  | HR (95% CI) | *P* |  | HR (95% CI) | *P* |
| Age | 1.60 (0.81, 3.08) | 0.18 |  | 0.84 (0.39, 1.85) | 0.67 |
| pT | 4.92 (2.39, 10.13) | <0.0001 |  | 2.35 (1.04, 5.32) | 0.04 |
| pN | 12.46 (5.84, 26.59) | <0.0001 |  | 6.76 (2.57, 17.76) | <0.0001 |
| Gleason score | 7.85 (1.88, 32.76) | 0.005 |  | 4.64 (1.08, 19.90) | 0.04 |
| CRYAB | 0.32 (0.15, 0.68) | 0.003 |  | 0.34 (0.15, 0.74) | 0.007 |

Age, between age≤62 and age>62; pT, pathologic tumor stage between T2 and T3-4; pN, pathologic regional lymph node metastasis, between N0 and N1; Gleason score, among Gleason score≤7 and >7; CRYAB, continuous CRYAB expression levels. HR, Hazard ratio; CI, confidence interval.
