# Supplementary material for: MiRNA-671-5p Promotes prostate cancer development and metastasis by targeting NFIA/CRYAB axis
Source: Cell Death Dis. 2020 Nov 3;11(11):949. doi: 10.1038/s41419-020-03138-w (PMC7642259; doi:10.1038/s41419-020-03138-w)
Supplement: Supplementary file 24 — Table S9 [file 41419_2020_3138_MOESM24_ESM.docx]

**Table S9.** Univariate and multivariate Cox regression analysis for biochemical recurrence-free survival in TCGA (NFIA)

|  | Univariate Cox regression analysis | |  | Multivariate Cox regression analysis | |
| --- | --- | --- | --- | --- | --- |
|  | HR (95% CI) | *P* |  | HR (95% CI) | *P* |
| Age | 1.15 (0.77, 1.73) | 0.50 |  | 0.88 (0.58, 1.32) | 0.53 |
| pT | 4.64 (2.47, 8.72) | <0.0001 |  | 2.93 (1.51, 5.69) | 0.002 |
| pN | 2.20 (1.42, 3.44) | <0.0001 |  | 1.01 (0.63, 1.61) | 0.98 |
| Gleason score | 4.33 (2.72, 6.88) | <0.0001 |  | 2.91 (1.76, 4.82) | <0.0001 |
| NFIA | 0.46 (0.30, 0.70) | <0.0001 |  | 0.61 (0.39, 0.95) | 0.03 |

Age, between age≤62 and age>62; pT, pathologic tumor stage between T2 and T3-4; pN, pathologic regional lymph node metastasis, between N0 and N1; Gleason score, among Gleason score≤7 and >7; NFIA, continuous NFIA expression levels. HR, Hazard ratio; CI, confidence interval.
