# Supplementary material for: MiRNA-671-5p Promotes prostate cancer development and metastasis by targeting NFIA/CRYAB axis
Source: Cell Death Dis. 2020 Nov 3;11(11):949. doi: 10.1038/s41419-020-03138-w (PMC7642259; doi:10.1038/s41419-020-03138-w)
Supplement: Supplementary file 25 — Table S10 [file 41419_2020_3138_MOESM25_ESM.docx]

**Table S10.** Univariate and multivariate Cox regression analysis for biochemical recurrence-free survival in TCGA (CRYAB)

|  | Univariate Cox regression analysis | |  | Multivariate Cox regression analysis | |
| --- | --- | --- | --- | --- | --- |
|  | HR (95% CI) | *P* |  | HR (95% CI) | *P* |
| Age | 1.15 (0.77, 1.73) | 0.50 |  | 0.91 (0.60, 1.37) | 0.66 |
| pT | 4.64 (2.47, 8.72) | <0.0001 |  | 2.89 (1.49, 5.63) | 0.002 |
| pN | 2.20 (1.42, 3.44) | <0.0001 |  | 1.10 (0.69, 1.75) | 0.70 |
| Gleason score | 4.33 (2.72, 6.88) | <0.0001 |  | 3.16 (1.91, 5.22) | <0.0001 |
| CRYAB | 1.15 (0.76, 1.72) | 0.51 |  | 1.19 (0.79, 1.80) | 0.40 |

Age, between age≤62 and age>62; pT, pathologic tumor stage between T2 and T3-4; pN, pathologic regional lymph node metastasis, between N0 and N1; Gleason score, among Gleason score≤7 and >7; CRYAB, continuous CRYAB expression levels. HR, Hazard ratio; CI, confidence interval.
