# Supplementary material for: MiRNA-671-5p Promotes prostate cancer development and metastasis by targeting NFIA/CRYAB axis
Source: Cell Death Dis. 2020 Nov 3;11(11):949. doi: 10.1038/s41419-020-03138-w (PMC7642259; doi:10.1038/s41419-020-03138-w)
Supplement: Supplementary file 26 — Table S11 [file 41419_2020_3138_MOESM26_ESM.docx]

Table S11. Basic information of included datasets

| ID | Subset | Data type | country | Sample type | n (ANT) | n (P) | n (M) | n (T) |
| --- | --- | --- | --- | --- | --- | --- | --- | --- |
| GSE21032 | GSE21036 | miRNA | USA | Human tissues | 28 | 99 | 14 | 113 |
|  | GSE21034 | mRNA | USA | Human tissues | 29 | 131 | 19 | 150 |
| TCGA | - | miRNA | USA | Human tissues | 48 | 442 | 3 | 482 |
|  | - | mRNA | USA | Human tissues | 52 | 456 | 3 | 498 |

Abbreviations: ID, identification; n, number; ANT, adjacent normal tissues; P, primary localized PCa tissues; M, metastatic PCa tissues.
