# Supplementary material for: MiRNA-671-5p Promotes prostate cancer development and metastasis by targeting NFIA/CRYAB axis
Source: Cell Death Dis. 2020 Nov 3;11(11):949. doi: 10.1038/s41419-020-03138-w (PMC7642259; doi:10.1038/s41419-020-03138-w)
Supplement: Supplementary file 27 — Table S12 [file 41419_2020_3138_MOESM27_ESM.docx]

Table S12. Primer information used in the study

| Primer | Sequence（5’ to 3’） |
| --- | --- |
| **For qRT-PCR** |  |
| NFIA Homo Forward | GCAGGCCCGAAAACGAAAATA |
| NFIA Homo Reverse | TTTGCCAGAAGTCGAGATGCC |
| CFL2 Homo Forward | AGAGGACCCCTACACATCTTTT |
| CFL2 Homo Reverse | CCAAGCCATTTACTTGCCACTC |
| RBMS3 Homo Forward | GGGGAACAGTTGAGTAAAACCA |
| RBMS3 Homo Reverse | ACAATTTTTCCATACGGTTGGCA |
| MPPED2 Homo Forward | ACAGGACTACTACCGTTTCCC |
| MPPED2 Homo Reverse | TTCACTGTTACCTCCGAATCTTG |
| CNN1 Homo Forward | CTGTCAGCCGAGGTTAAGAAC |
| CNN1 Homo Reverse | GAGGCCGTCCATGAAGTTGTT |
| CRYAB Homo Forward | AGGTGTTGGGAGATGTGATTGA |
| CRYAB Homo Reverse | GGATGAAGTAATGGTGAGAGGGT |
| ACTC1 Homo Forward | GTACCCTGGTATTGCTGATCG |
| ACTC1 Homo Reverse | CCTCATCGTACTCTTGCTTGCT |
| CLU Homo Forward | CCAATCAGGGAAGTAAGTACGTC |
| CLU Homo Reverse | CTTGCGCTCTTCGTTTGTTTT |
| TGM4 Homo Forward | TGAATCAGGACAACGCCGTTT |
| TGM4 Homo Reverse | GTGGTAGGATTGTAGGGGCTG |
| GADPH Homo Forward | ACAACTTTGGTATCGTGGAAGG |
| GADPH Homo Reverse | GCCATCACGCCACAGTTTC |
| **For PCR amplification in ChIP** |  |
| Site #1 Homo Forward | CAATCTGGCCCTTTCACAAT |
| Site #1 Homo Reverse | TCTCCCCTAGCAGGCTGTAA |
| Site #2 Homo Forward | AACAGATCACCCATGTGCAA |
| Site #2 Homo Reverse | CACCTTCCAGAACCATCCAT |
| Site #3 Homo Forward | GGGTGAGGGAAATCTGTCAA |
| Site #3 Homo Reverse | AGGGAGGGAGGTCACTTGTT |
